# Oncolytic peptide LTX-315 induces anti-pancreatic cancer immunity by targeting the ATP11B-PD-L1 axis

**DOI:** 10.1136/jitc-2021-004129

**Published:** 2022-03-14

**Authors:** Tianyu Tang, Xing Huang, Gang Zhang, Minghao Lu, Zhengtao Hong, Meng Wang, Junming Huang, Xiao Zhi, Tingbo Liang

**Affiliations:** 1 Department of Hepatobiliary and Pancreatic Surgery, The First Affiliated Hospital, Zhejiang University School of Medicine, Hangzhou, People's Republic of China; 2 Zhejiang Provincial Key Laboratory of Pancreatic Disease, The First Affiliated Hospital, Zhejiang University School of Medicine, Hangzhou, People's Republic of China; 3 Zhejiang Clinical Research Center of Hepatobiliary and Pancreatic Diseases, Hangzhou, People's Republic of China; 4 The Innovation Center for the Study of Pancreatic Diseases of Zhejiang Province, Hangzhou, People's Republic of China; 5 Cancer Center, Zhejiang University, Hangzhou, People's Republic of China

**Keywords:** immunotherapy, programmed cell death 1 receptor, tumor microenvironment, gastrointestinal neoplasms, drug therapy, combination

## Abstract

**Background:**

LTX-315 is an oncolytic peptide deriving from bovine lactoferrin, with the ability to induce cancer immunogenic cell death. However, the mechanism used by LTX-315 to trigger the antitumor immune response is still poorly understood. The expression of programmed cell death ligand 1 (PD-L1) largely determines the efficacy and effectiveness of cancer immunotherapies targeting this specific immune checkpoint. This study aimed to demonstrate the potential effect and mechanism of LTX-315 in PD-L1 inhibition-induced anti-pancreatic cancer immunity.

**Methods:**

Both immunodeficient and immunocompetent mouse models were used to evaluate the therapeutic efficacy of monotherapy and combination therapy. Flow cytometry and immunohistochemistry were used to assess the immune microenvironment. Multiomic analysis was used to identify the potential target and down-streaming signaling pathway. Both in-house tissue microarray and open accessed The Cancer Genome Atlas data sets were used to evaluate the clinical relevance in pancreatic cancer prognosis.

**Results:**

LTX-315 treatment inhibited PD-L1 expression and enhanced lymphocyte infiltration in pancreatic tumors. ATP11B was identified as a potential target of LTX-315 and a critical regulator in maintaining PD-L1 expression in pancreatic cancer cells. As regards the mechanism, ATP11B interacted with PD-L1 in a CKLF-like MARVEL transmembrane domain containing 6 (CMTM6)-dependent manner. The depletion of ATP11B promoted CMTM6-mediated lysosomal degradation of PD-L1, thus reactivating the immune microenvironment and inducing an antitumor immune response. The significant correlation among ATP11B, CMTM6, and PD-L1 was confirmed in clinical samples of pancreatic cancer.

**Conclusions:**

LTX-315 was first identified as a peptide drug inducing PD-L1 downregulation via ATP11B. Therefore, LTX-315, or the development of ATP11B-targeting drugs, might improve the efficacy of cancer immunotherapy.

## Background

Pancreatic cancer is one of the leading causes of cancer-related death worldwide.^1^ It is characterized by histologic, genetic, and molecular heterogeneity, and is generally diagnosed at an advanced stage, with a high risk of metastasis and a high tendency of early recurrence.^2 3^ The 5-year survival rate among patients with pancreatic ductal adenocarcinoma (PDAC) is less than 10%, due to its aggressive biological behavior and limited therapeutic strategies.^4–6^ Radical resection followed by adjuvant chemotherapy is considered the first-line treatment in patients at an early stage.^4^ However, only 15%–25% of the patients present at the clinic with the resectable form of this tumor; thus, the majority is not suitable for surgery.^4 7^ For patients with advanced pancreatic cancer, chemotherapy with FOLFIRINOX or nab-paclitaxel plus gemcitabine, is usually used to improve the outcome of patients.^5 6 8 9^ However, the response rate of the first-line chemotherapy regimen is only 30%–35% and the problem of a consequent high rate of acquired resistance is remaining.^6^ Currently, immunotherapy represented by the use of neutralizing antibodies targeting immune checkpoints, particularly programmed cell death protein 1 (PD-1) and its ligand 1 (PD-L1), has become the most promising approach with significant clinical benefits in a variety of malignancies.^10 11^ However, the efficacy of PD-1/PD-L1-targeted immunotherapy in pancreatic cancer is still limited, since only a few patients show an improvement of their clinical status.^12 13^

Tumor response to PD-1/PD-L1 blockade therapy is considered largely, even not totally, contingent on pre-existing T cell immunity in tumor microenvironment (TME), which is associated with the mutation burden of tumors, the expression level of immune checkpoints, the infiltration degree of T-lymphocytes, and the clonality diversity of T cell receptors.^14^ However, PDAC usually lacks pre-existing T cell immunity under pathophysiological condition, at least partially due to its low immunogenicity and immunosuppressive TME.^14^ Increasing evidence suggests that the combination therapy has the potential to synergically activate T cell-mediated antitumor immunity, improving the therapeutic efficacy of PD-1/PD-L1 blockade in pancreatic cancer. For instance, hepatocyte growth factor receptor MET-specific small molecule inhibitors induce the infiltration and activation of CD8 +T cells in pancreatic cancer, and significantly enhance the antitumor effects of PD-1/PD-L1 blockade in preclinical mouse models.^15^ Similarly, the inhibition of NIMA-related kinase 2 also sensitizes PD-L1-blocking antibodies, synergically stimulating the anti-pancreatic cancer immune response.^16^ In addition, targeting Pin1 with clinically available agents causes complete elimination or sustained remissions of PDAC by synergizing with gemcitabine and anti-PD-1 in diverse model systems.^17^ In clinical, PD-1/PD-L1 blockade therapy has also been tried to combine with other treatment, such as chemoradiotherapy and cancer vaccine, for improving the therapeutic efficacy of pancreatic cancer.^18^ Taken together, these findings indicate that combination strategy holds promise for overcoming the low effectiveness in PD-1/PD-L1-targeted pancreatic cancer immunotherapy. Therefore, it is urgently needed to establish highly effective combination therapy, incorporating PD-1/PD-L1 blockade drugs with other potent partner agents to maximize the clinical benefits for patients with pancreatic cancer.^5 19 20^

Cationic anti-microbial peptides (CAPs) are naturally occurring molecules used by various species against bacteria and foreign pathogens as part of the innate immune system.^21^ Interestingly, CAPs have also been found to possess potential cytotoxic activity against tumors, together with limited side effects.^21 22^ LTX-315 is a cationic peptide derived from bovine lactoferrin and composed of nine amino acids that exert strong oncolytic effects on human cancer cells whereas are well tolerated by normal cells.^23^ This peptide has thereby been investigated in multiple malignancies in preclinical animal models.^24–26^ Intriguingly, the LTX-315-mediated oncolysis triggers an antitumor immune response through inducing tumor immunogenic cell death, characterized by the exposure or release of diverse danger-associated molecular patterns (DAMPs) including calreticulin, ATP, and high mobility group box 1.^27 28^ This process culminates in the recruitment of antigen-presenting cells into the TME, ultimately priming a tumor-targeting cytotoxic T lymphocyte-dependent immune attack.^22^ LTX-315 has also been observed to deplete intratumoral myeloid-derived suppressor cells (MDSCs) and CD4 +CD25+FOXP3+regulatory T cells, two populations with potent immunosuppressive effects.^22^ Moreover, the intratumoral injection of LTX-315 activates natural killer (NK) cell-dependent immunity.^24 25^ Recently, Spicer *et al* reported the first phase I clinical trial on LTX-315 in 27 patients with solid tumors.^29^ Consistent with preclinical findings, the intratumoral administration of LTX-315 causes a significant change in the TME, and the security is relatively high. A total of 14 patients underwent pre-treatment and post-treatment biopsies, and 12 (86%) showed increased infiltration of CD8 +T cells. Twelve (12/27) patients achieved immune-related stable disease and the remaining experienced immune-related progression disease as the best response. Although shrinkage of distant non-injected lesions up to 82% in a single lesion was observed, none with LTX-315 treatment showed an immune-related response according to Intratumoral Response Evaluation Criteria. Interestingly, LTX-315 in combination with anti-PD-1 antibody was observed to induce a potent immune response and long-term immune memory protection in melanoma.^30^ Nevertheless, the therapeutic potential of LTX-315 and its feasibility in combination with PD-1/PD-L1 blockade for pancreatic cancer are largely unknown. Additionally, the detailed mechanism underlying the synergy of LTX-315 and PD-1/PD-L1 blockade is still not fully understood.

This work showed that LTX-315 treatment enhanced the tumor infiltration of lymphocytes and synergized with PD-L1/PD-1 blockade in pancreatic cancer therapy. ATP11B was identified as a potential target of LTX-315 and a novel regulator in maintaining the expression of PD-L1 in pancreatic cancer. Overall, this work conceptually highlighted the effect of the combination immunotherapy based on the oncolytic peptide and immune checkpoint blockade and further demonstrated the critical role of ATP11B in PD-L1 regulation.

## Materials and methods

### Antibodies, inhibitors, and agents

The following antibodies were used for immunoprecipitation/immunoblotting: rabbit anti-ATP11B (SAB3500528, Sigma-Aldrich), rabbit anti-ATP11B (13 672–1-AP, Proteintech), mouse anti-β actin (ab8226, Abcam), rabbit anti-CKLF-like MARVEL transmembrane domain containing 6 (CMTM6) (HPA026980, Sigma-Aldrich), rabbit anti-CMTM6 (55829, Cell Signaling Technology), rabbit anti-CMTM6 (DF3944, Affinity), mouse anti-PD-L1 (66 248–1-Ig, Proteintech), and rabbit anti-PD-L1 (13684, Cell Signaling Technology). The following antibodies were used for immunohistochemical (IHC) staining: rabbit anti-CD8 (98941, Cell Signaling Technology), rabbit anti-PD-L1 (64988, Cell Signaling Technology), rabbit anti-ATP11B (13 672–1-AP, Proteintech), rabbit anti-PD-L1 (13684, Cell Signaling Technology), and rabbit anti-CMTM6 (DF3944, Affinity). The following antibodies were used for flow cytometry: Brilliant Violet 785 mouse anti-CD45 (109839, BioLegend), BV605 rat anti-mouse CD45 (567459, BD BioSciences), Brilliant Violet 605 rabbit anti-CD8a (100743, BioLegend), PE-Cy7 rat anti-CD8α (561097, BD BioSciences), FITC rabbit anti-CD3 (100203, BioLegend), APC-Cy7 rat anti-CD4 (552051, BD BioSciences), PE-CF594 rat anti-CD11b (562399, BD BioSciences), BV605 rat anti-ly6C (563011, BD BioSciences), FITC rat anti-ly6G (561105, BD BioSciences), PerCP-Cy5.5 rat anti-CD11C (560584, BD BioSciences), BV510 rat anti-CD24 (747717, BD BioSciences), PerCP/Cyanine5.5 mouse anti-Granzyme B (396412, BioLegend), PE mouse anti-CD274 (124308, BioLegend), PE anti-TNF-α (12-7423-41, eBioscience), APC anti-IFN-γ (17-7319-82, eBioscience), and APC rabbit anti-CD274 (124312, BioLegend). The following inhibitors and agents were used: MG132 (S2619, Selleck), aloxistatin (S7393, Selleck), pepstatin A (S7381, Selleck), CDDP (S1166, Selleck), and LTX-315 (S8199, Selleck). All compounds were used at the dilution suggested by the manufacturers’ instructions.

### ELISA

Culture supernatants of BXPC-3 and SW1990 cells with or without LTX-315 treatment were individually collected at the indicated time points. Specific sandwich ELISA kits were used to detect the concentrations of the secreted interleukin (IL)-6 (1110602, DAKEWE) and IL-8 (1110802, DAKEWE), according to the manufacturer’s protocol.

### Bioinformatic analysis

TISIDB (http://cis.hku.hk/TISIDB) is a web portal that explores the interaction between tumor and immune system by the integration of multiple heterogeneous datum types, including genomics, transcriptomics, and clinical data of 30 non-hematologic cancer types from The Cancer Genome Atlas (TCGA).^31^ Therefore, in the current study, TISIDB was used to explore the correlation between certain investigated genes and the abundance of the tumor-infiltrating lymphocytes (TILs) or immunoinhibitors in multiple cancer types. The immune-related signatures of the 28 investigated TIL types were collected according to the protocol published by Charoentong *et al*.^32^ Each Spearman correlation between certain investigated genes and selected TIL types or immunoinhibitors in an individual cancer type was integrated into the indicated heatmap. GEPIA2 (http://gepia2.cancer-pku.cn, V.2) is an open-access online tool to analyze the RNA expression of 9736 tumors and 8587 normal samples from the TCGA and the GTEx projects, using a standard processing pipeline.^33^ Thus, GEPIA2 was used to explore the profiles of differentially expressed genes and the prognostic significance of certain investigated genes, as well as the correlation among the genes. The statistical difference in the expression of certain investigated genes was evaluated by one-way analysis of variance (ANOVA). The survival analysis according to the expression of certain investigated genes was performed using the Kaplan-Meier method with a 50% (median) cut-off value for both the low and high expression group. The hypothesis was tested using the log-rank test (Mantel-Cox test), the HR was calculated using the cox proportional hazards regression model, and a value of p<0.05 was used as the threshold. The pairwise gene expression correlation among different genes was analyzed by the Spearman correlation analysis, and the results with a value of p<0.05 were considered statistically significant.

### Multiomic analyses

The RNA-sequencing was performed as follows: after the extraction of the total RNA, RNA purity was evaluated by the NanoPhotometer spectrophotometer (IMPLEN, California, USA) and RNA integrity by the RNA Nano 6000 Assay Kit of the Bioanalyzer 2100 system (Agilent Technologies, California, USA). Sequencing libraries were generated using NEBNext UltraTM RNA Library Prep Kit for Illumina (NEB, USA) according to the manufacturer’s protocol. The library quality was assessed on the Agilent Bioanalyzer 2100 system. The library preparations were sequenced on an Illumina platform after cluster generation. The raw data (raw reads) in fastq format were first processed through in-house Perl scripts for quality control, and Hisat2 (V.2.0.5) was selected as the mapping tool. After read mapping, the number of reads mapped to each gene were counted by FeatureCounts V.1.5.0-p3. Subsequently, the Fragments Per Kilobase of transcript sequence per Millions (FPKM) of each gene were calculated according to the length of the gene and the read counts mapped to this gene. The analysis of the differentially expressed genes in the selected two groups was performed using the DESeq2 R package (V.1.16.1), and the genes with an adjusted p value <0.05 found by DESeq2 were considered as differentially expressed. In addition, gene set enrichment analysis (GSEA) was performed, and gene sets with a p value <0.05 according to DAVID and containing at least 10 genes per pathway were reported. The Tandem mass tag (TMT) proteomic analysis was performed as follows: after extraction of the total proteins, the protein concentration of each sample was calculated using the Bradford protein quantitative kit. Afterwards, 120 µg protein of each sample were mixed with 3 µL trypsin (1 µg/µL) and 500 µL triethylamonium bicarbonat (TEAB) buffer (50 mM), and the mix was digested at 37°C overnight. The digested sample was mixed with an equal volume of 1% formic acid and centrifuged at 12,000 g for 5 min at room temperature. The supernatant was collected and desalted using a C18 desalting column and the desalted peptides were labeled using a specific labeling reagent (100 µL TEAB buffer 0.1 M and 41 µL acetonitrile dissolved in TMT labeling reagent). All labeled samples were mixed in an equal volume, desalted, and lyophilized. The mobile phase A (2% acetonitrile, with a pH adjusted to 10.0 by ammonium hydroxide) and B (98% acetonitrile, with a pH adjusted to 10.0 by ammonium hydroxide) were used to develop a gradient elution. A Rigol L3000 HPLC system with a C18 column was used to fraction the samples. The eluates were monitored at UV 214 nm, and they were divided in a tube per minute and combined into 10 fractions. All fractions were dried under vacuum and reconstituted in 0.1% (v/v) formic acid in water. Shotgun proteomics was performed using an EASY-nLCTM 1200 UHPLC system (Thermo Fisher) coupled with a Q Exactive HF mass spectrometer (Thermo Fisher) operating in the data-dependent acquisition mode, and finally, the raw data of MS detection was developed. The identification and quantification of proteins were performed using the software Proteome Discoverer V.2.2 (Thermo Fisher Scientific). The quantified proteins were statistically analyzed by the Mann-Whitney test, and the values p<0.05 and |log2FC|>1.5 resulting from the comparison of the proteins between experimental and control groups were used to define the differentially expressed proteins.

### Cell lines and cell culture

BXPC-3, SW1990, Hepa1-6, and HEK293T cell lines were purchased from the American Type Culture Collection (ATCC, Manassas, Virginia, USA). The KPC cell line deriving from the LSL-Kras G12D/+; LSL-Trp53 R172H/+; Pdx1-Cre mouse model was kindly provided by Professor Raghu Kalluri (Department of Cancer Biology, Division of Basic Sciences, MD Anderson Cancer Center, Houston, Texas, USA). BXPC-3, SW1990, HEK293, and KPC were cultured in 1640 medium (SH30027.0, Life Sciences) and Hepa1-6 were cultured in Dulbecco's modified eagle medium supplemented with 10% fetal bovine serum (FBS) and 1% Pen/Strep (SH30022.01, Life Sciences). All cell lines were incubated at 37°C under 5% CO_2_. Mycoplasma contamination was routinely evaluated by PCR.

### Ethics approval and consent to participate

All procedures performed in this study involving human participants were in accordance with the ethical standards of the Ethics Committee of the First Affiliated Hospital of Zhejiang University School of Medicine, as well as approved and supervised by the same Ethics Committee. The procedures were also in accordance with the 1964 Helsinki declaration and its later amendments or comparable ethical standards. All individuals provided written informed consent to participate in this study. Animal care and use in all animal experiments were performed in accordance with the applicable guidelines of the Animal Ethics Committee of the First Affiliated Hospital of Zhejiang University School of Medicine.

### Animal care and use

Immunodeficient Balb/c nude mice and immunocompetent C57BL/6 mice were obtained from the Nanjing Biomedical Research Institute of Nanjing University and were maintained under specific pathogen-free conditions in the experimental animal center of the First Affiliated Hospital, College of Medicine, Zhejiang University. The animal experiments were approved by the Institutional Animal Care and Use Committee of the First Affiliated Hospital, College of Medicine, Zhejiang University. As regards the monotherapy and combination therapy, 8-week-old male Balb/c nude mice and C57BL/6 mice were treated with a subcutaneous (*s.c*.) injection of 5×10^5^ KPC cells/Hepa1-6 cells on their right flank. The tumor volume was assessed as length×width^2^×0.5. The antitumor treatment started when the palpable tumors achieved a volume of 50–100 mm^3^. Tumor-bearing mice were randomly divided into groups with similar average tumor volumes before the treatment. LTX-315 was administered by an intratumoral injection at a dose of 0.5 mg/mouse, two times a week. Anti-mouse PD-L1 (Bio X Cell, West Lebanon, New Hampshire, USA) was intraperitoneally injected at a dose of 200 µg/mouse, three times a week. Anti-mouse PD-1 (Bio X Cell) was intraperitoneally injected at a dose of 120 µg/mouse, three times a week. Tumor growth was evaluated by measuring the volume with a caliper. At the end of the experiment, mice were weighed before the sacrifice, and tumors were collected, weighed, and divided into different parts for subsequent analysis. As regards the pretreatment with LTX-315, 8-week-old male Balb/c nude mice and C57BL/6 mice were individually treated witan an *s.c*. injection of 3×10^5^ KPC cells on their right flank with or without pretreatment with LTX-315 for 24 hours. The time of palpable tumors occurrence was recorded. As regards the ATP11B depletion treatment, KPC-ATP11B KO cells and KPC-WT cells at a density of 5×10^5^ in 25 µL PBS mixed with Matrigel were separately injected into the pancreas of 8-week-old male Balb/c nude mice and C57BL/6 mice. At the end of the experiment, mice were weighed before the sacrifice, and tumors were collected, weighed, and divided into different parts for subsequent analysis.

### Cell proliferation, viability, and colony formation

As regards cell proliferation, cells were seeded in six-well plates in triplicate at a density of 1×10^4^ cells/well (day 0) in 3 mL normal growth medium, which was changed every 2 days. The cell number in each well at the indicated time points was calculated under light microscopy and compared. As regards cell viability, 2.5×10^3^ of each of the indicated cells were seeded in 96-well microplates in triplicates in 200 µL normal growth medium per well. The treated cells were incubated with a mixture of 10 µL CCK-8 solution and 90 µL serum-free medium at the indicated time points for 2 hours at 37°C under 5% CO_2_. The absorbance was measured at 450 nm in a microplate reader and the percentage of viable cells was averaged and calculated for each well. As regards colony formation, cells were seeded in six-well plates in triplicate at a density of 500–1000 (day 0) in 3 mL 1640 medium supplemented with 10%–20% FBS per well. Cells were incubated at 37°C under 5% CO_2_ for 2 weeks, and the growth medium was replaced every 2 days. At the end of the incubation, cells were fixed with 4% paraformaldehyde for 1 hour and then stained with 0.1% crystal violet for 2 hours. Pictures of each replicate were taken after washing with distilled water. The colony number was calculated and averaged.

### Overexpression and depletion of genes in cell lines

Cells at 60%–80% confluence were transiently transfected with human and mouse Flag-ATP11B DNA (Obio Technology, Shanghai), human and mouse Flag-CMTM6 DNA (Obio Technology, Shanghai), and human myc-PD-L1 (Obio Technology, Shanghai), using jetPRIME reagent (Polyplus) according to the manufacturer’s instructions and were selected by puromycin or hygromycin. KPC, BXPC-3, and SW1990 cells were stably transfected or co-transfected with human ATP11B Double Nickase Plasmid (sc-411692-NIC, Santa Cruz), and ATP11B Double Nickase Plasmid (sc-429296-NIC, Santa Cruz) and KPC and SW1990 were stably transfected with CMTM6 Double Nickase Plasmid (sc-412381-NIC, Santa Cruz) and CMTM6 Double Nickase Plasmid (sc-426447-NIC, Santa Cruz) according to the manufacturer’s instructions. After transfection, cells were diluted and isolated before being seeded again to ensure the single-cell clone formation. The efficiency of depletion was evaluated by western blot analysis.

### Western blot and immunoprecipitation

Cells were lysed in RIPA lysis buffer (P0013B, Beyotime Biotechnology) and 1 mM phenylmethanesulfonyl fluoride (ST505, Beyotime Biotechnology) on ice for 30 min. The lysate was centrifuged at 12,000 g for 15 min, and protein concentration in the supernatant was measured by the BCA Protein Assay Reagent Kit (P0012, Beyotime Biotechnology). The supernatant was boiled in 1×NuPAGE LDS Sample Buffer (Thermo Fisher Scientific) for 5 min and subjected to 8%–12% SDS-PAGE for the separation of the proteins, followed by their transfer to a nitrocellulose membrane (N66485, PALL) or a PVDF membrane (IPFL00010, Millipore). The membrane was blocked with 5% skim milk in TBST for 1 hour, treated with the indicated antibodies, and incubated overnight. The membrane was washed three times with TBST, treated with the HRP-conjugated antibodies, and visualized using ChemiScopeTouch (Clinx Science Instruments). As regards immunoprecipitation, cells were lysed in IP lysing buffer (P0013, Beyotime Biotechnology), protease inhibitor cocktail (B14001, Bimake), and phosphatase inhibitor cocktail (B15001, Bimake) for 60 min on ice and centrifuged at 12,000 g for 15 min. The supernatant was incubated with the indicated antibodies, anti-Flag magnetic beads (B26101, Bimake) or anti-Myc magnetic beads (B26301, Bimake) for 4–6 hours at 4°C, followed by the addition of 15 µL protein A/G magnetic beads (B23201, Bimake) and incubated for additional 2 hours at 4°C (only for IP with unconjugated antibodies). The samples were washed three times with washing buffer, boiled in 1×NuPAGE LDS Sample Buffer (NP0007, Thermo Fisher Scientific) for 5 min, and immunoblotted as described above. ChemiScopeTouch (Clinx Science Instruments) was used for visualization, and β–actin was used as the loading control.

### Analysis of infiltrated immune-cell fractions

The total RNA of the tumors was extracted using TRIzol reagent. RNA purity was evaluated by the NanoPhotometer spectrophotometer (IMPLEN, California, USA) and RNA integrity by Agilent 2100 Bioanalyzer (Agilent Technologies, California, USA). Sequencing libraries were generated using TruSeq Stranded messenger RNA LT Sample Prep Kit (Illumina, California, USA) according to the manufacturer’s protocol. Sequencing libraries were generated using an Illumina HiSeq X Ten platform (NEB, USA). The raw data (raw reads) in fastq format were first processed using Trimmomatic, and the clean reads were obtained after the low-quality reads were removed. After read mapping, the number of reads was mapped to each gene using HISAT2. The number of reads mapped to each gene was counted by HTSeq-count. Subsequently, the FPKM of each gene were calculated based on the length of the gene and read counts mapped to this gene. GSEA was performed, and gene sets with a p value <0.05 according to DAVID and containing at least 10 genes per pathway were reported. CIBERSORTx (https://cibersortx.stanford.edu/) is an online analytical tool designed by the Alizadeh Lab and Newman Lab to estimate the composition of cell types in a tumor with a complex cell population based on gene expression data.^34 35^ In this study, CIBERSORT was used to quantify the abundance of immune infiltrated cells in the tumor treated with LTX-315 based on leukocyte signature matrix 22 (LM22) and RNA transcripts data. Normalized data were uploaded and analyzed using the deconvolution algorithm with 1000 permutations. A threshold of the p value less than 0.05 was recommended.

### Protein purification and in vitro pull down

The flag-ATP11B protein was purified using anti-Flag magnetic beads (B26101, Bimake), and then incubated with the flag peptide to release the protein from the anti-Flag magnetic beads. The eluate was then incubated with purified Myc-PD-L1 using anti-Myc magnetic beads (B26301, Bimake) for an additional 2 hours at 4°C. The samples were washed three times with washing buffer, boiled in 1×NuPAGE LDS Sample Buffer (NP0007, Thermo Fisher Scientific) for 5 min, and subjected to western blot.

### IHC staining

IHC staining was performed to evaluate CD8 and PD-L1 in formalin-fixed paraffin-embedded tissue sections of mouse tumors. Slides (3–5 µm thick) were baked at 68°C for 80 min, deparaffinized and rehydrated, treated with the primary antibodies against the proteins indicated above, and incubated at 4°C overnight. Next, the slides were incubated with a biotin-conjugated secondary antibody at room temperature for 30 min and the proteins were visualized using the Diaminobenzidine Chromogen Kit (BDB2004, Biocare). The slides were counterstained with diluted hematoxylin and visualized under a light microscope. ZEN Connect software (Zeiss) was used to capture the representative images. PDAC tissue microarray was obtained from the Department of Hepatobiliary and Pancreatic Surgery, the First Affiliated Hospital, School of Medicine, Zhejiang University, containing 156 carcinoma tissue samples and created by the Wuhan Servicebio technology. The involved patients gave their written informed consent. IHC staining of the PDAC tissue microarray was performed by the Wuhan Servicebio technology. The proteins labeled by the IHC staining (CD8, ATP11B, CMTM6, and PD-L1) were quantified using 3DHISTECH QuantCenter V.2.1 software.

### Flow cytometry

Tumors were harvested at the end of the in vivo experiments. The tumor was cut into little pieces, digested using a mixture of 1640 medium containing 2% FBS and 3 mM CaCl_2_, collagenase (17104019, Thermo Fisher Scientific), Dispase (17105041, Gibco), and DNase (10 µg/mL) (D5025, Sigma-Aldrich) and incubated at 37°C for 1 hour under shaking at 200 rpm. The single-cell suspension was filtered using 40 µm cell strainers (08-771-1, Thermo Fisher Scientific), and red blood cells were removed using lysis buffer. The dead cells were recognized using the invitrogen LIVE/DEAD fixable dead cell stain. As regards the cell surface staining, cells were first blocked using CD16/CD32 antibody for 30 min, then incubated at 4°C and CD45, CD3, CD8, CD326, CD11b, CD11c, CD24, MHCII, Ly6G, Ly6C, and PD-L1 proteins in the cell surface were stained. As regards the intracellular staining, cells were fixed and permeabilized using the fixation/permeabilization solution kit (555028, BD BioSciences), and stained for intracellular Granzyme B, tumor necrosis factor (TNF)-α, and interferon (IFN)-γ. All samples were analyzed using a CytoFLEX (Beckman Coulter) and data were analyzed by FlowJo software (V.10.4).

### Statistical analysis

Statistical analysis was performed using SPSS V.18.0 and GraphPad Prism V.7.0. Results were presented as mean±SD of at least three independent experiments. Two groups were compared using the Student’s t-test, while multiple groups were compared using one-way ANOVA. The survival difference was analyzed using the log-rank test or Gehan-Breslow-Wilcoxon test. The correlation between different genes was obtained using Pearson’s correlation analysis. A two-tailed test with p<0.05 was considered statistically significant.

### Data availability

All data generated or analyzed during this study are included in this article and its online supplemental information files. Additional information is available from the corresponding author on reasonable request.

10.1136/jitc-2021-004129.supp1 Supplementary data

## Results

### LTX-315 enhances PD-L1-targeted pancreatic cancer immunotherapy

**Figure 1:**
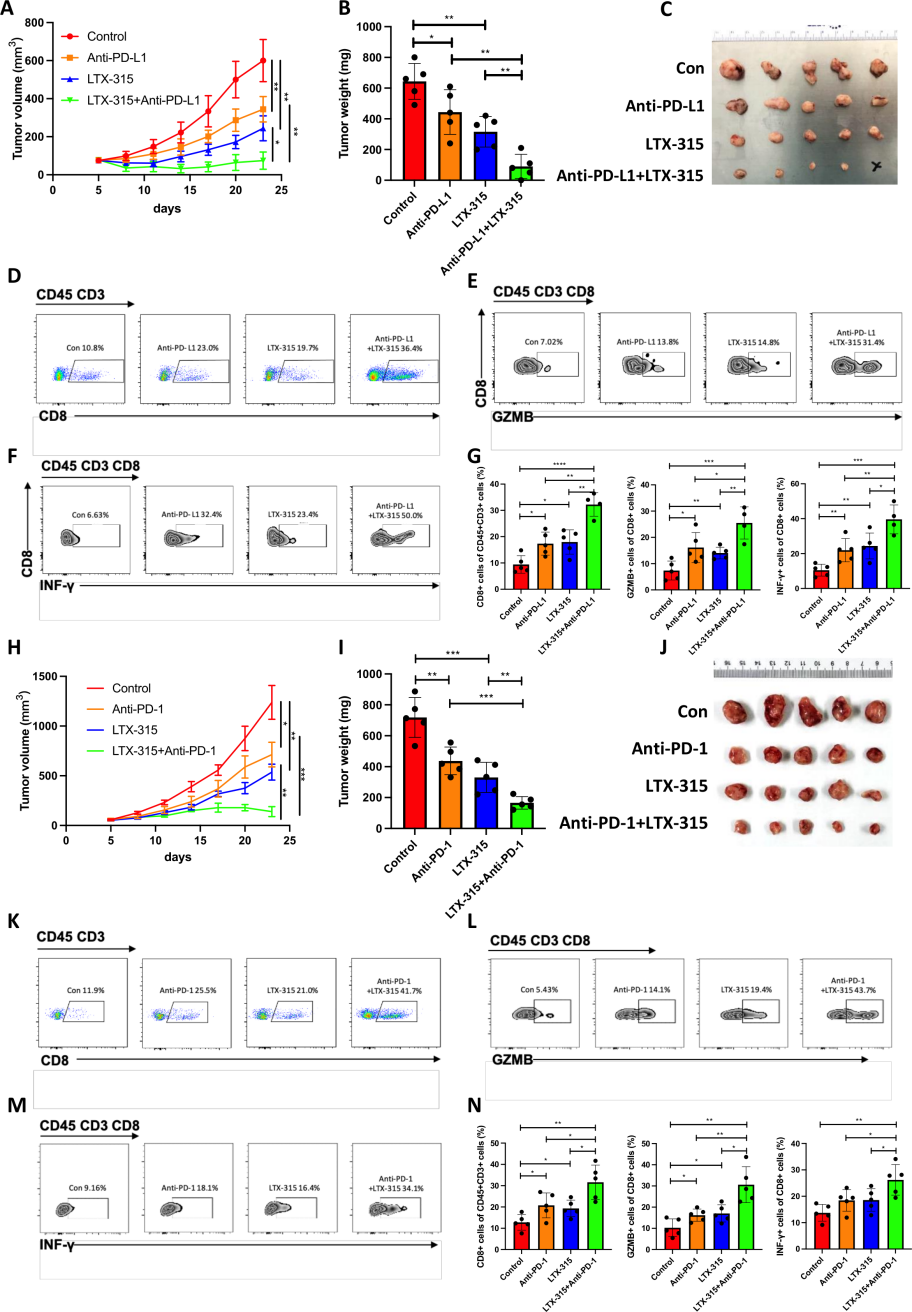
LTX-315 synergizes with PD-L1-targeted therapy in pancreatic cancer. (A–G) The combination of LTX-315 and PD-L1-targeted therapy inhibits pancreatic tumor growth. KPC cells were *s.c*. inoculated into immunocompetent mice (n=5). Growth curves of tumors were recorded at the indicated time points (A). Representative images of tumor weight (B) and tumors (C) were individually recorded at the experimental endpoints. Representative images of TILs are individually shown (D–F) and further quantified (G). (H–N) The combination of LTX-315 and PD-1-targeted therapy inhibits pancreatic tumor growth. KPC cells were *s.c*. inoculated into immunocompetent mice (n=5). Growth curves of tumors were recorded at the indicated time points (H). Representative images of tumor weight (I) and tumors (J) were individually recorded at the experimental endpoints. Representative images of TILs are individually shown (K–M) and further quantified (N). Results are presented as mean±SD of one representative experiment. *p<0.05, **p<0.01, ***p<0.001 by a two-tailed t-test; INF, interferon; NS, not significant; PD-L1, programmed cell death ligand 1; s.c., subcutaneous; TILs, tumor-infiltrating lymphocytes.

LTX-315 was first combined with PD-L1/PD-1 blockade in pancreatic cancer to evaluate whether this combination could exert a synergistic therapeutic effect. The treatment with LTX-315 and/or anti-PD-L1 antibody on C57BL/6 mice with palpable KPC tumors revealed that the combination of the two treatments resulted in more significant inhibition of tumor growth compared with the inhibition in the control group, or in the mice treated only with LTX-315 or only with anti-PD-L1 antibody (figure 1A–C). Moreover, the quantity of infiltrated CD8 +T cells (figure 1D, G), activated Granzyme B+T cells (figure 1E, G) and IFN-γ+T cells (figure 1F, G) was increased in tumors treated with the combined therapy. The increased infiltration of CD8 +T cells in the combined treatment was confirmed by IHC staining (online supplemental figure S1A, B). Furthermore, the combination of LTX-315 and PD-L1 blockade resulted in the longest survival time of mice (online supplemental figure S1C). Similarly, the treatment with LTX-315 and/or anti-PD-1 antibody on C57BL/6 mice with palpable KPC tumors also showed a significant synergistic therapeutic effect. The combination of the two treatments resulted in significant inhibition of tumor growth, especially in the combination group (figure 1H–J). The infiltrated CD8 +T cells (figure 1K, N), activated Granzyme B+T cells (figure 1L, N), and IFN-γ+T cells (figure 1M, N) were also increased in the tumors treated with the combined therapy. The synergistic therapeutic effect was also evaluated in a different experimental model, such as C57BL/6 mice with palpable Hepa1-6 tumors treated with LTX-315 and/or anti-PD-L1 antibody. As expected, the tumor growth was largely inhibited in the combination group (online supplemental figure S2A–C). In addition, both CD8 +T cells and activated Granzyme B+T cells were significantly increased in the combination group, which was consistent with the results in the pancreatic cancer in vivo model (online supplemental figure S2D, E). Taken together, these results suggested that the combined treatment of LTX-315 with PD-1/PD-L1 blockage therapy synergistically inhibited tumor growth and enhanced the infiltration of lymphocytes into the tumor and local immune response.

### LTX-315 reshapes the tumor immune microenvironment and induces anti-pancreatic cancer immunity

**Figure 2:**
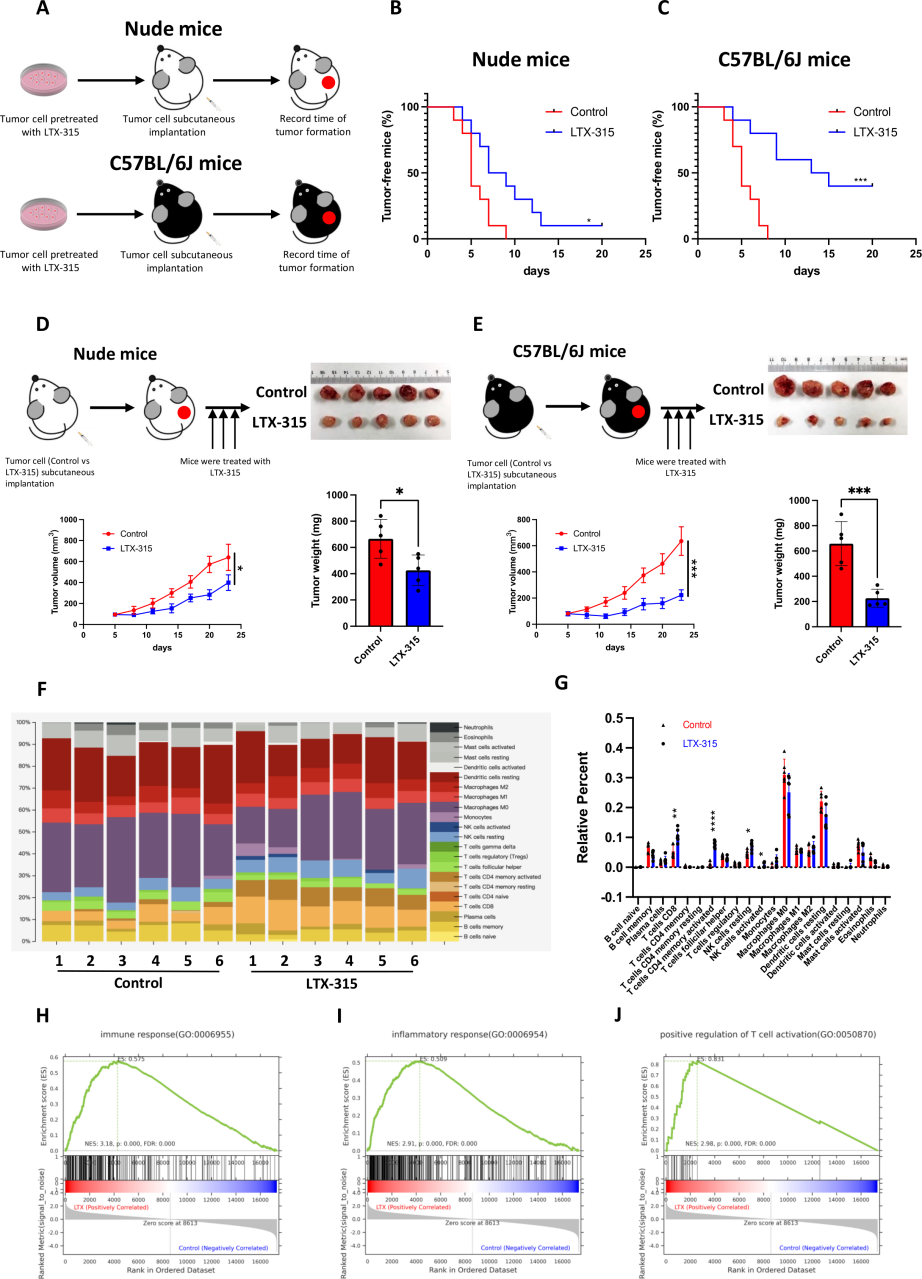
LTX-315 reshapes the immune-microenvironment in pancreatic cancer. (A–C) LTX-315 pretreatment suppresses pancreatic tumorigenesis largely through the immune system. KPC cells with or without LTX-315 pretreatment were separately and *s.c*. inoculated into the immunodeficient and immunocompetent mice (n=10). Schematic protocols are shown as indicated (A). Tumor incidence in the immunodeficient (B) and immunocompetent (C) mice individually recorded at the indicated times. (D–E) LTX-315 treatment inhibited pancreatic tumor growth largely through the immune system. KPC cells were separately and *s.c*. inoculated into immunodeficient and immunocompetent mice (n=6). Growth curves of the tumors; representative images and weight of tumors in the immunodeficient (D) and immunocompetent (E) mice individually recorded at the indicated time points. (F–J) Representative images of the infiltrated immune cell proportion (F) and further quantification of immune cell infiltration induced by LTX-315 using CIBERSORT (G). Top regulated immune pathways identified by gene set enrichment analysis (H–J). Results are presented as mean±SD of one representative experiment. *p<0.05, **p<0.01, ***p<0.001 by a log-rank test or a two-tailed t-test; NS, not significant; s.c., subcutaneous.

KPC cells, with or without LTX-315 pretreatment, were separately *s.c*. injected into nude mice and C57BL/6 mice (figure 2A), revealing that LTX-315 pretreatment resulted in a lower tumor incidence and longer tumor-free survival in immunocompetent mice. Indeed, only 10% of the nude mice remained tumor-free at 20 days after inoculation of the KPC cells pretreated with LTX-315 (figure 2B), while 40% of the C57BL/6 mice remained tumor-free after the same number of days after KPC inoculation (figure 2C). Moreover, both immunodeficient nude mice and immunocompetent C57BL/6 mice bearing KPC tumors were individually treated with an intratumor injection of LTX-315 to further investigate the immunological dependence of LTX-315-mediated pancreatic cancer inhibition in vivo. As expected, LTX-315 treatment exerted a stronger inhibitory effect on tumor growth in C57BL/6 mice compared with the effect exerted in nude mice (figure 2D, E), indicating the involvement of the immune system in the action of LTX-315. Tumors from C57BL/6 mice were further subjected to transcriptomic quantification analysis to understand the effect of LTX-315 on the tumor immune microenvironment. Cibersort was applied to estimate the alteration in the immune composition. Interestingly, CD8 +T cells, activated CD4 +memory cells, as well as resting and activated NK cells were significantly upregulated in the LTX-315 treated group (figure 2F, G, and online supplemental figure S3A). In addition, GSEA revealed a significant upregulation of genes involved in the antitumor immune response (figure 2H, I) and T cell activation (figure 2J). Collectively, these results indicated that LTX-315 reshaped the TME and inhibited pancreatic tumor growth in an immune system-dependent manner.

### LTX-315 downregulates PD-L1 expression

**Figure 3:**
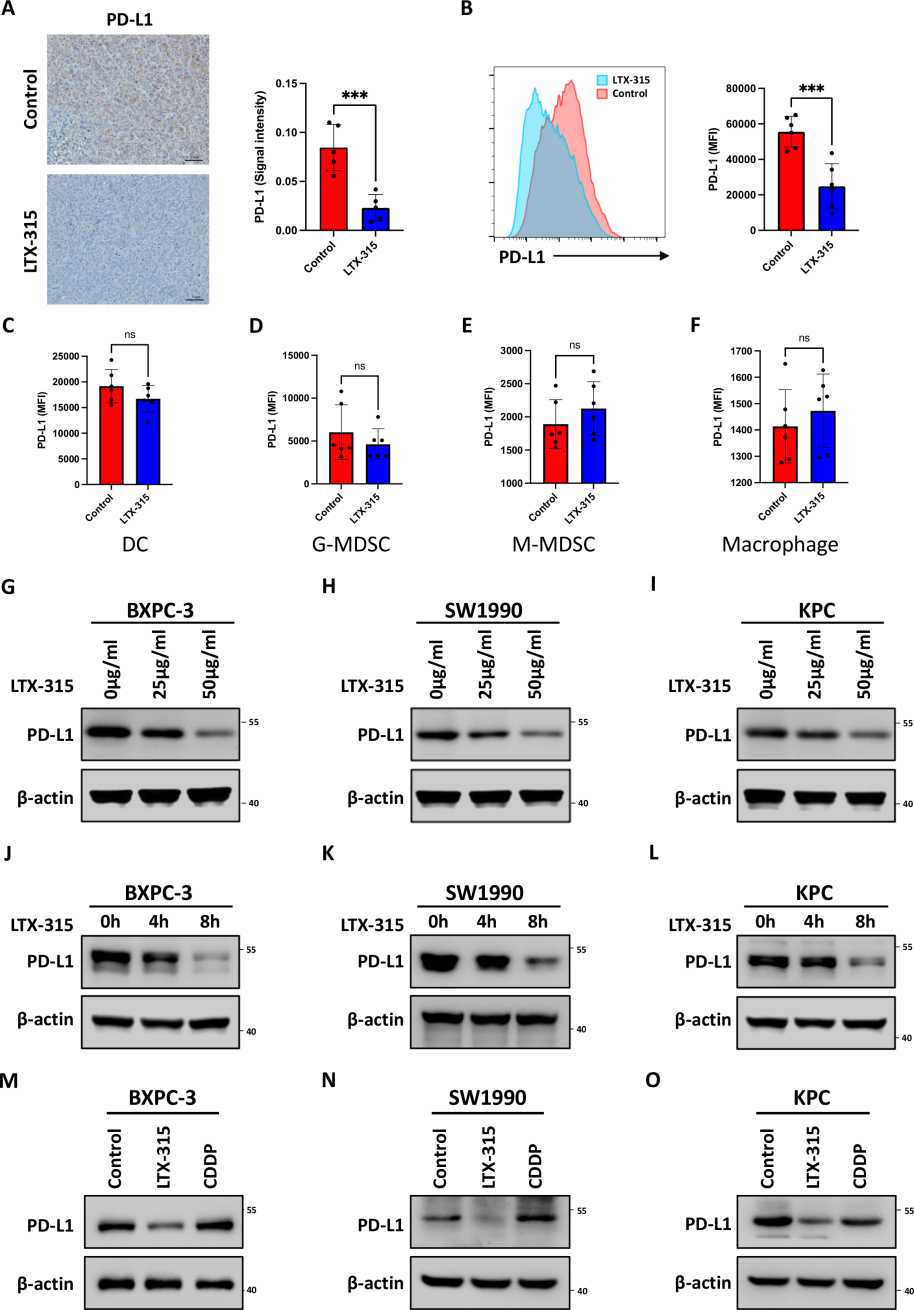
LTX-315 downregulates PD-L1 expression in pancreatic cancer. (A) Representative images and further quantification of PD-L1 IHC staining in the immunocompetent mice treated with LTX-315. (B–F) Representative images and further quantification of flow cytometry analysis of PD-L1 expression in tumor cells (B), dendritic cells (C), granulocytic-MDSCs (D), monocytic-MDSCs (E), and macrophages (F) in tumors collected from immunocompetent mice. (G–I) LTX-315 inhibition of PD-L1 expression in a dose-dependent manner. PD-L1 expression in BxPC-3 (G), SW1990 (H), and KPC (I) cell treated with LTX-315 at increasing concentrations, individually analyzed by western blotting. (J–L) Inhibition of PD-L1 expression by LTX-315 in a time-dependent manner. PD-L1 expression in BxPC-3 (J), SW1990 (K), and KPC (L) cell treated with LTX-315 at increasing time points, individually analyzed by western blotting. (M–O) Inhibition of PD-L1 expression by LTX-315 regardless of the effect of cell death. PD-L1 expression in BxPC-3 (M), SW1990 (N), and KPC (O) cells treated with LTX-315 and CDDP, individually analyzed by western blotting. Results are presented as mean±SD of one representative experiment. *p<0.05, **p<0.01, ***p<0.001 by a two-tailed t-test; DC, dendritic cell; IHC, immunohistochemical; MDSCs, myeloid-derived suppressor cells; NS, not significant; PD-L1, programmed cell death ligand 1.

Considering the synergistic therapeutic effect of LTX-315 with PD-1/PD-L1 targeted therapy and the significant role played by PD-L1/PD-1 in tumor immune escape, the involvement of LTX-315 in the regulation of PD-L1 expression was further investigated. Intriguingly, IHC staining revealed that PD-L1 expression was significantly reduced in tumors treated with LTX-315 (figure 3A). According to previous reports, PD-L1 is expressed on different types of cells, including tumor cells, macrophages, MDSCs, and dendritic cells. Therefore, PD-L1 expression on different cell populations was further determined using flow cytometry. The results showed that PD-L1 was significantly downregulated in tumor cells (figure 3B and online supplemental figure S4A), while the alteration of PD-L1 in other cell populations did not reach a statistical significance (figure 3C–F and online supplemental figure S4B). In addition, PD-L1 on BXPC-3, SW1990, and KPC cells was significantly downregulated by LTX-315 in a dose-dependent and time-dependent manner (figure 3G–L). Since LTX-315 causes tumor cell lysis and eventually death, the next step was to evaluate whether the downregulation of PD-L1 was induced by the cell lytic effect of LTX-315. Therefore, CDDP was used as an agent associated with non-immunogenic cell death in a further test. However, pancreatic cancer cell lines BXPC-3 (figure 3M), SW1990 (figure 3N), and KPC (figure 3O) showed that PD-L1 was significantly decreased when the cells were treated with LTX-315 compared with the control and CDDP group. Taken together, these results indicated that LTX-315 downregulated PD-L1 in pancreatic cancer, an effect that was independent of its cell lytic effect.

### Identification of downstream signaling pathways and effector molecules of LTX-315

**Figure 4:**
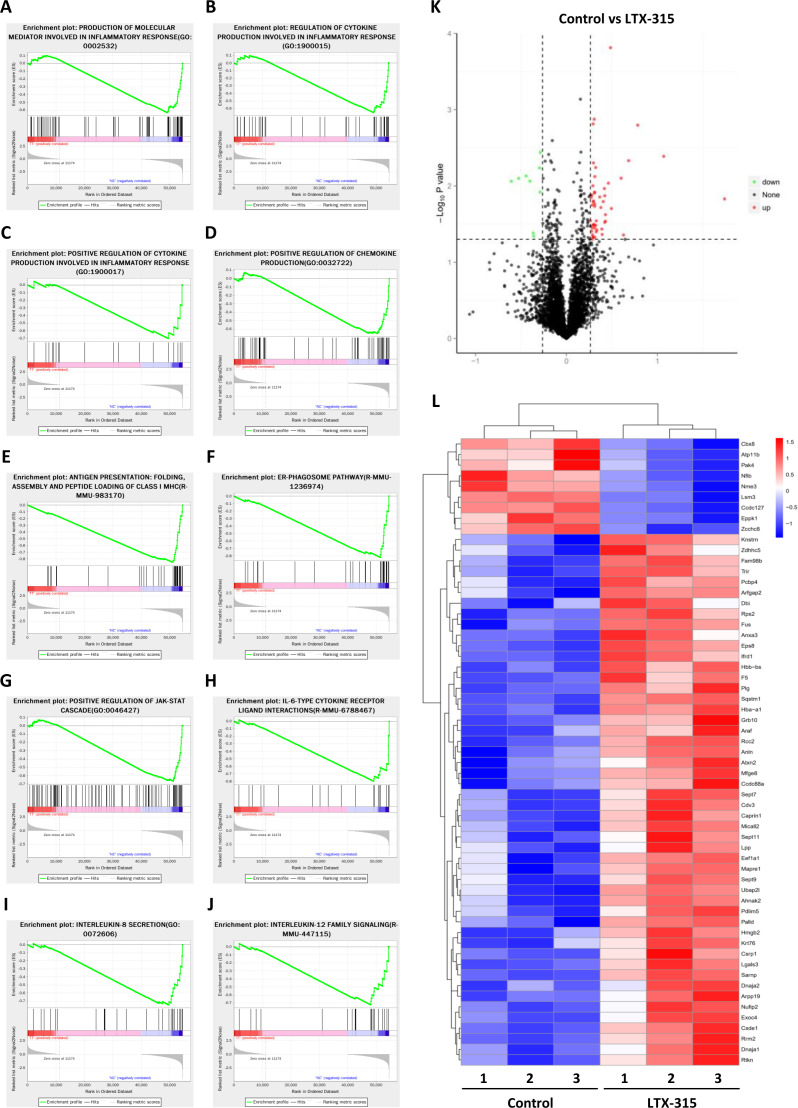
Identification of the downstream signaling pathways and effector molecules of LTX-315 by multiomic analysis. (A–J) Top regulated pathways in transcriptomics analysis of KPC cells with or without LTX-315 treatment by gene set enrichment analysis, including the production of molecular mediators involved in inflammatory response (A), regulation of cytokine production involved in inflammatory response (B), positive regulation of cytokine production involved in inflammatory response (C), positive regulation of chemokine production (D), antigen presentation, folding assembly and peptide loading of class I MHC (E), ER-phagosome pathway (F), positive regulation of JAK-STAT cascade (G), IL-6-type cytokine receptor ligand interactions (H), interleukin-8 secretion (I), and interleukin-12 family signaling (J). (K) Volcano plot of TMT proteomics analysis displaying the pairwise comparison between KPC cells with or without LTX-315 treatment. (L) TMT proteomics analysis reveals differentially expressed proteins between KPC cells with or without LTX-315 treatment. Heat map of top-regulated proteins by LTX-315. IL, interleukin; TMT, tandem mass tag.

Transcriptomic and proteomic quantitative analysis was performed in KPC cells with or without LTX-315 treatment to investigate the underlying mechanism used by LTX-315 to inhibit PD-L1. Our results revealed that LTX-315 could induce a significant upregulation or downregulation of a wide variety of genes (online supplemental figure S5A). GSEA indicated a significant alteration in multiple immune pathways by LTX-315, including but not limited to, the production of molecular mediators involved in the inflammatory response (figure 4A), the regulation of cytokine production involved in the inflammatory response (figure 4B), the positive regulation of cytokine production involved in the inflammatory response (figure 4C), the positive regulation of chemokine production (figure 4D), antigen presentation, folding assembly and peptide loading of class I MHC (figure 4E), ER-phagosome pathway (figure 4F), positive regulation of JAK-STAT cascade (figure 4G), IL-6-type cytokine receptor ligand interactions (figure 4H), IL-8 secretion (figure 4I), and IL-12 family signaling cytokine regulation (figure 4J). The alteration of IL-6 and IL-8 secretion was also confirmed using ELISA in BXPC-3 (online supplemental figure S6A) and SW1990 (online supplemental figure S6B) treated with LTX-315. Further proteomic analysis revealed that LTX-315 treatment on cells induced an alteration of 57 proteins (figure 4K, L). Taken together, these results suggested that LTX-315 had a profound impact on pancreatic cancer cells, which might be correlated with its regulatory effect on PD-L1 expression.

### ATP11B is a novel suppressor of PD-L1 in pancreatic cancer immunity

**Figure 5:**
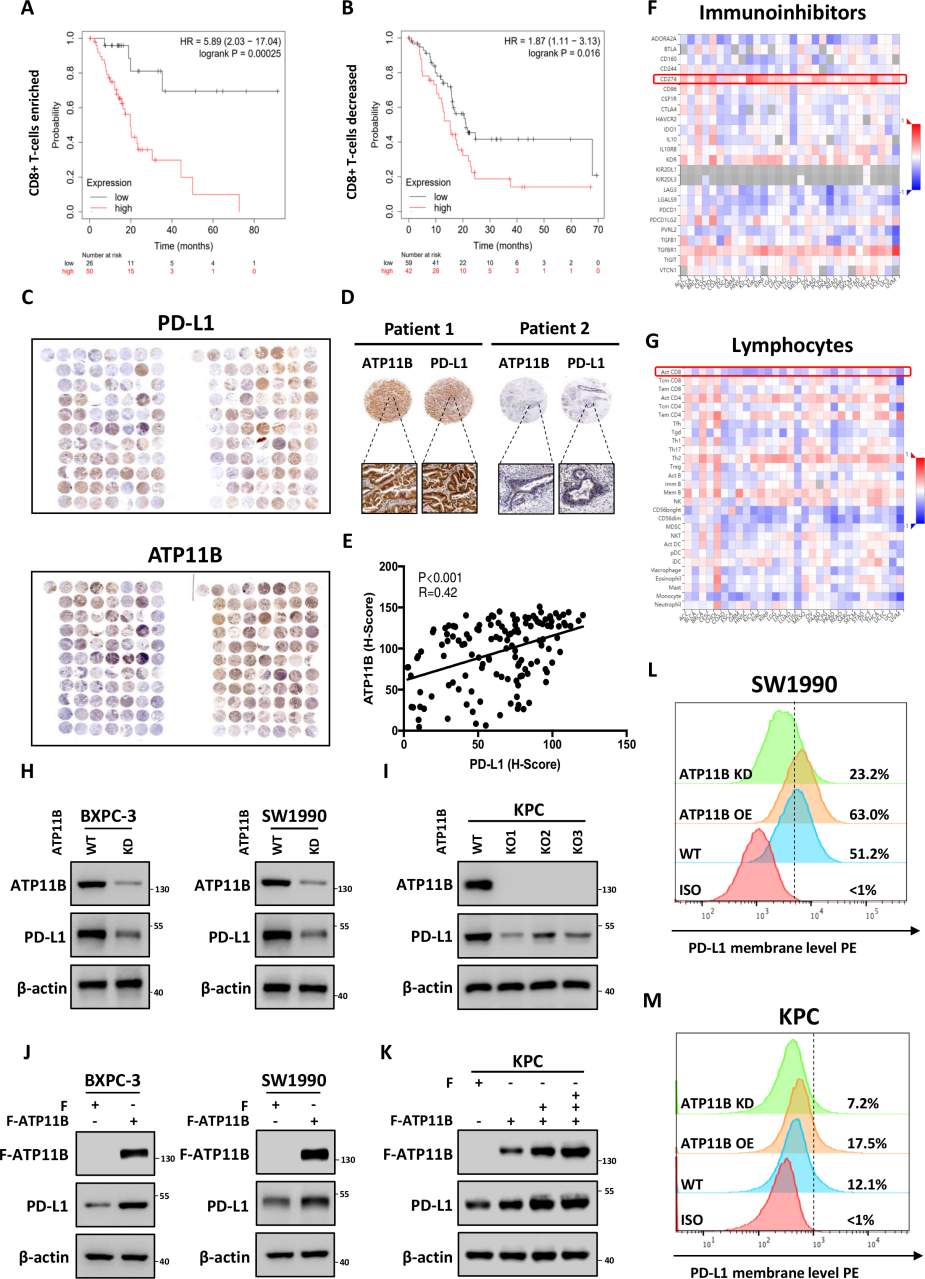
LTX-315-reduced ATP11B is a potential regulator of cancer immunity and PD-L1 expression. (A–B) Overall survival analysis in CD8 +T cell enriched (A) and CD8 +T cell decreased (B) patients with pancreatic cancer with low or high ATP11B expression. (C–E) Positive correlation of ATP11B with PD-L1 in pancreatic cancer. Representative images (C−D) and statistical results (E) of ATP11B and PD-L1 IHC staining in pancreatic cancer tissue microarray. (F–G) Correlation between the expression of ATP11B and abundance of immunoinhibitors (F) as well as tumor-infiltrating lymphocytes (G) across multiple human cancers, as analyzed on the The Cancer Genome Atlas database. (H–M) Maintenance of PD-L1 expression by ATP11B in multiple pancreatic cancer cell lines. Western blotting of PD-L1 expression in ATP11B KD/KO pancreatic cancer cell lines (H−I) and cell lines overexpressing ATP11B (J−K). Flow cytometry showing PD-L1 expression in ATP11B KD/KO pancreatic cancer cell lines and cell lines overexpressing ATP11B (L−M). IHC, immunohistochemical; KD, knockdown; KO, knockout; PD-L1, programmed cell death ligand 1; WT, wild type.

The immunological correlation and prognostic value of the significantly altered proteins identified by the proteomic analysis were investigated based on TCGA data sets. Patients were stratified into immune ‘hot’ tumor (rich in CD8 +T cell infiltration) and ‘cold’ tumor (low in CD8 +T cell infiltration) groups according to CD8 +T cell infiltration. Intriguingly, ATP11B, the second most downregulated protein, showed an immunological prognostic relevance in pancreatic patients. ATP11B is one of the transmembrane ATPases reported as involved in both the maintenance of the immune-suppressive microenvironment and the regulation of cellular vesicle trafficking and membrane protein expression. The results showed that the high ATP11B expression was associated with a poor prognosis in both groups, since the survival rate was significantly decreased in CD8 +T cell-rich patients with high ATP11B expression, suggesting that the influence of ATP11B on pancreatic cancer prognosis largely depended on T cell priming (figure 5A, B). The functional validation revealed that ATP11B was downregulated by LTX-315 in a time-dependent and dose-dependent manner (online supplemental figure S7A–F). Tissue microarray analysis of the 156 pancreatic cancer samples further revealed that ATP11B and PD-L1 were indeed positively correlated (figure 5C–E). Notably, the integrated bioinformatic analysis performed on multiple cancers showed that ATP11B expression was positively correlated with the expression of various immune-related modulators, including PD-L1, but negatively correlated with the activated CD8 +T cells (figure 5F, G, online supplemental figure S8A–D), suggesting the critical role of ATP11B in cancer immune resistance. Next, ATP11B knockout (KO) and knockdown (KD) pancreatic cancer cell lines, or cell lines overexpressing ATP11B, were generated to evaluate the regulatory relationship between ATP11B and PD-L1 in pancreatic cancer. The results showed that both ATP11B KD and KO resulted in a decreased expression of PD-L1 (figure 5H, I) in SW1990, BXPC-3, and KPC cells, while its overexpression in the above cells was followed by the accumulation of PD-L1 (figure 5J, K). A similar alteration was observed in the membrane PD-L1 expression (figure 5L, M). These last findings indicated that ATP11B regulated PD-L1 expression in pancreatic cancer.

### ATP11B interacts with PD-L1 in a CMTM6-dependent manner

**Figure 6:**
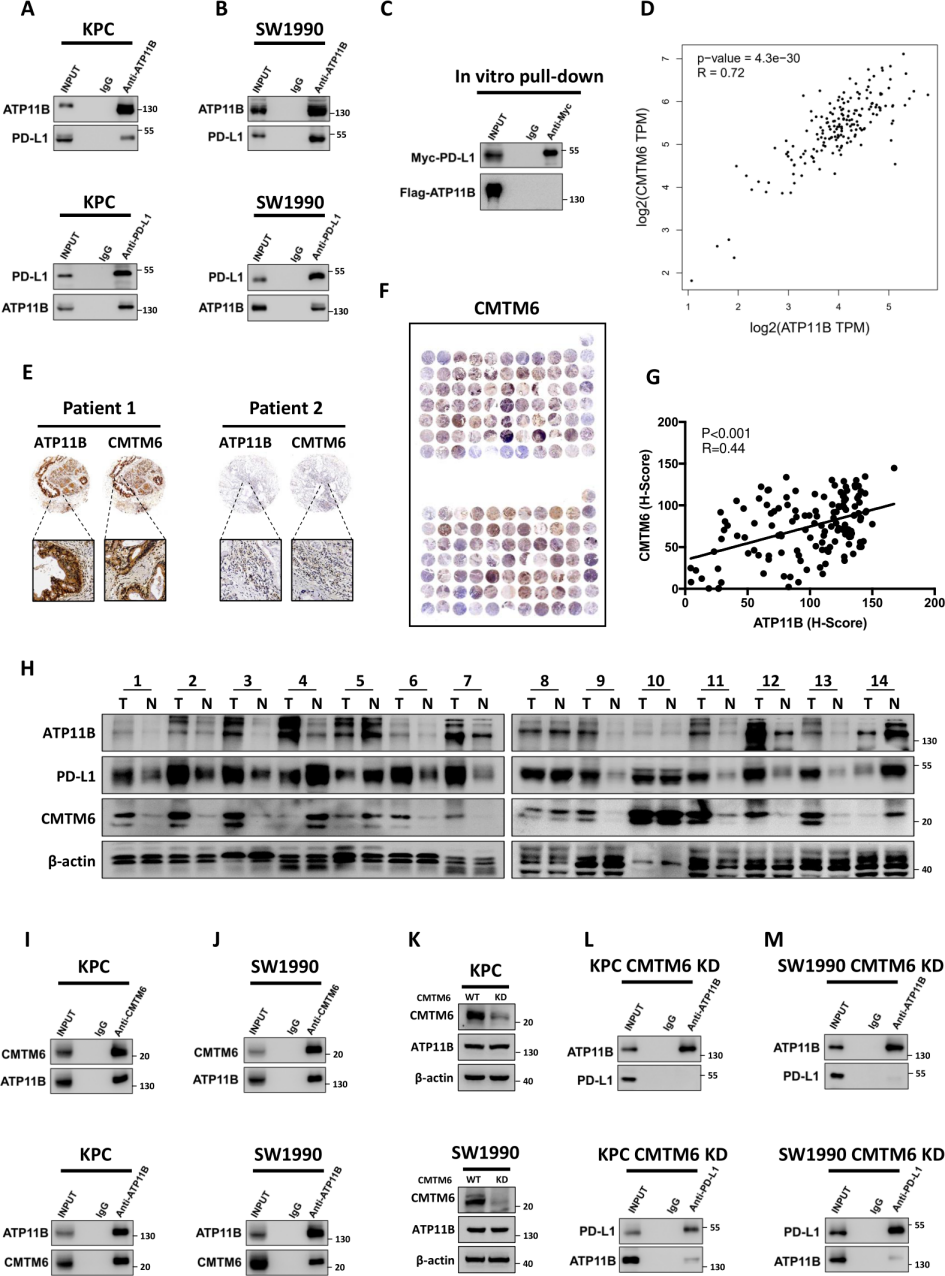
ATP11B interacts with PD-L1 in a CMTM6-dependent manner. (A–B) ATP11B interaction with PD-L1 in vivo. Cell lysates from KPC and SW1990 cells were separately subjected to immunoprecipitation and western blotting using the indicated antibodies. (C) Lack of binding of ATP11B to PD-L1 in vitro. FLAG-tagged ATP11B and MYC-tagged PD-L1 purified from 293 T cell lysates and subjected to immunoprecipitation after co-incubation. (D) Positive correlation of ATP11B with CMTM6. Correlation of ATP11B and CMTM6 analyzed according to the pancreatic data sets of The Cancer Genome Atlas. (E–G) Representative images (E–F) and statistical results (G) of CMTM6 and ATP11B immunohistochemical staining in pancreatic cancer tissue microarray. (H) Western blotting of ATP11B, CMTM6, and PD-L1 expression in paired clinical tissue samples. (I–M) Interaction of ATP11B with PD-L1 in pancreatic cancer in a CMTM6-dependent manner. (I−J) cell lysates from KPC and SW1990 cells were separately subjected to immunoprecipitation and western blotting using the indicated antibodies. (K) Western blotting of ATP11B expression in pancreatic cancer cell lines after CMTM6 knockdown. (L–M) Cell lysates from CMTM6-KD KPC (L) and CMTM6-KD SW1990 (M) separately subjected to immunoprecipitation and western blotting using the indicated antibodies. CMTM6, CKLF-like MARVEL transmembrane domain containing 6; PD-L1 programmed cell death ligand 1.

The presence of a potential interaction between ATP11B and PD-L1 was evaluated to discover how ATP11B regulates the expression of PD-L1. Intriguingly, although ATP11B endogenously interacted with PD-L1 in KPC and SW1990 cells (figure 6A, B), such interaction could not be obtained in vitro using purified recombinant ATP11B and PD-L1 proteins (figure 6C). Previous studies showed that the CMTM6 acts as an adaptor protein, binding to PD-L1 to mediate its endosomal recycling, thereby protecting PD-L1 from lysosomal degradation to maintain its quantity on the cell membrane.^36 37^ Interestingly, ATP11B and CMTM6 in pancreatic cancer were positively correlated (figure 6D), as revealed by the bioinformatic analysis using TCGA data sets. The positive correlation between ATP11B and CMTM6 was further confirmed by tissue microarray (figure 6E–G). Moreover, CMTM6 was negatively associated with activated CD8 +T cells (online supplemental figure S9A), while positively correlated with PD-L1 expression in pancreatic cancer and many others (online supplemental figure S9B). In addition, CMTM6 possessed a significant prognostic relevance only in CD8 +T cell-rich patients, indicating that CMTM6 might affect T cell priming like ATP11B (online supplemental figure S9C, D). Furthermore, paired clinical tissue samples were further used to confirm the correlation among ATP11B, CMTM6, and PD-L1 (figure 6H). The above findings might us speculate that ATP11B regulated PD-L1 expression via CMTM6. Indeed, further experiments revealed the endogenous interaction between ATP11B and CMTM6 in KPC (figure 6I) and SW1990 (figure 6J) cells. More importantly, CMTM6 KD had no impact on the expression of ATP11B (figure 6K), but it caused the disruption of the ATP11B-PD-L1 interaction to a large extent (figure 6L, M). Taken together, these results suggested that CMTM6 might be involved in the ATP11B action on PD-L1 in pancreatic cancer.

### ATP11B prevents lysosomal degradation of PD-L1 via CMTM6

**Figure 7:**
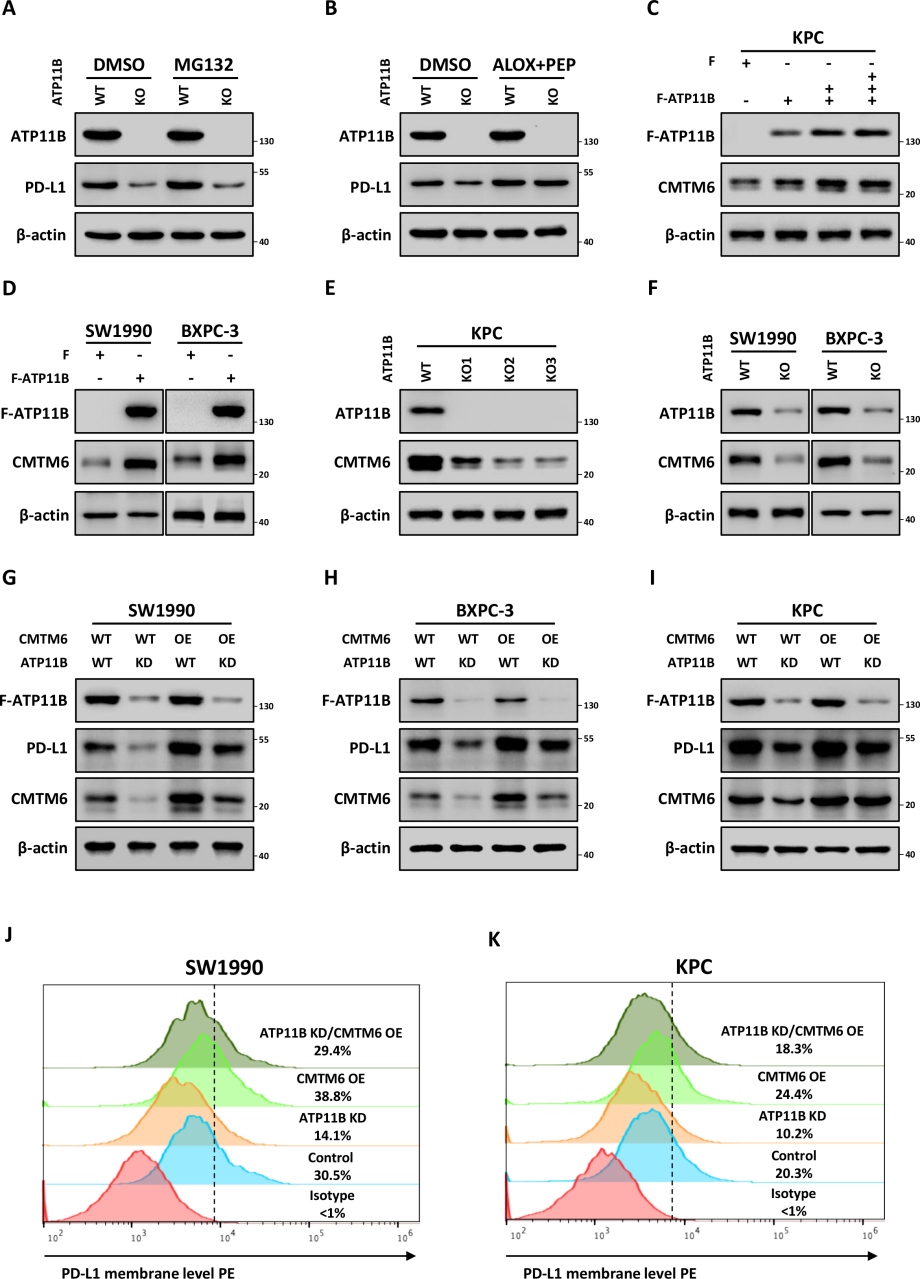
ATP11B prevents lysosomal degradation of PD-L1 through the interaction with CMTM6. (A–B) regulation of PD-L1 expression by ATP11B via lysosome-mediated degradation. (A) PD-L1 expression was analyzed by western blotting in WT and ATP11B KO KPC treated with or without the proteasome inhibitor MG132. (B) PD-L1 expression was analyzed by western blotting in WT and ATP11B KPC KO treated with or without the lysosome inhibitor aloxistatin and pepstatin A. (C–F) regulation of CTMT6 expression by ATP11B in pancreatic cancer. CMTM6 expression was analyzed by western blotting in pancreatic cancer cell lines overexpressing ATP11B (C–D) and ATP11B KD/KO pancreatic cell lines (E–F). (G–K) Rescues of the decrease of PD-L1 by CMTM6 overexpression caused by ATP11B kD. Western blotting (G–I) and flow cytometry (J–K) analysis of PD-L1 expression in WT/ATP11B kD pancreatic cancer cell lines with or without CMTM6 overexpression. CMTM6, CKLF-like MARVEL transmembrane domain containing 6; KD, knockdown; KO, knockout; PD-L1, programmed cell death ligand 1; WT, wild type.

Wild type (WT) and ATP11B KO KPC cells were used to investigate the critical role of CMTM6 in the ATP11B-PD-L1 axis. These cells were treated with the proteasome inhibitor MG132 or lysosome inhibitors aloxistatin +pepstatin A, respectively. The results revealed that ATP11B depletion-induced PD-L1 downregulation could not be restored by MG132 (figure 7A), but it was almost totally restored by the treatment with aloxistatin +pepstatin A (figure 7B), suggesting that ATP11B maintained PD-L1 expression by the inhibition of its degradation by lysosomes. ATP11B overexpression caused the increase of CMTM6 in KPC, SW1990, and BXPC-3 cells (figure 7C, D), while ATP11B KO or KD resulted in the decrease of its expression (figure 7E, F). Moreover, the overexpression of CMTM6 could reverse ATP11B depletion-induced downregulation of PD-L1 protein (figure 7G–I), which was in line with the results of the alteration of PD-L1 expression on the cell membrane (figure 7J, K). The potential clinical significance of the ATP11B-CMTM6 complex in pancreatic cancer evaluated by TCGA data sets revealed that ATP11B was significantly correlated with tumor stage and pathologic grade (online supplemental figure S10A, B), while CMTM6 was associated with the pathologic grade (online supplemental figure S10C, D). Furthermore, both ATP11B and CMTM6 were significantly upregulated in PDAC compared with their expression in normal tissue (online supplemental figure S11A, B) and also associated with the prognosis of patients (online supplemental figure S11C, D). Collectively, these findings demonstrated that ATP11B maintained PD-L1 expression through the prevention of its lysosomal degradation mediated by CMTM6, suggesting that the aberrantly expressed ATP11B might be a potential target to combat pancreatic cancer.

### ATP11B depletion activates anti-pancreatic cancer immunity

**Figure 8:**
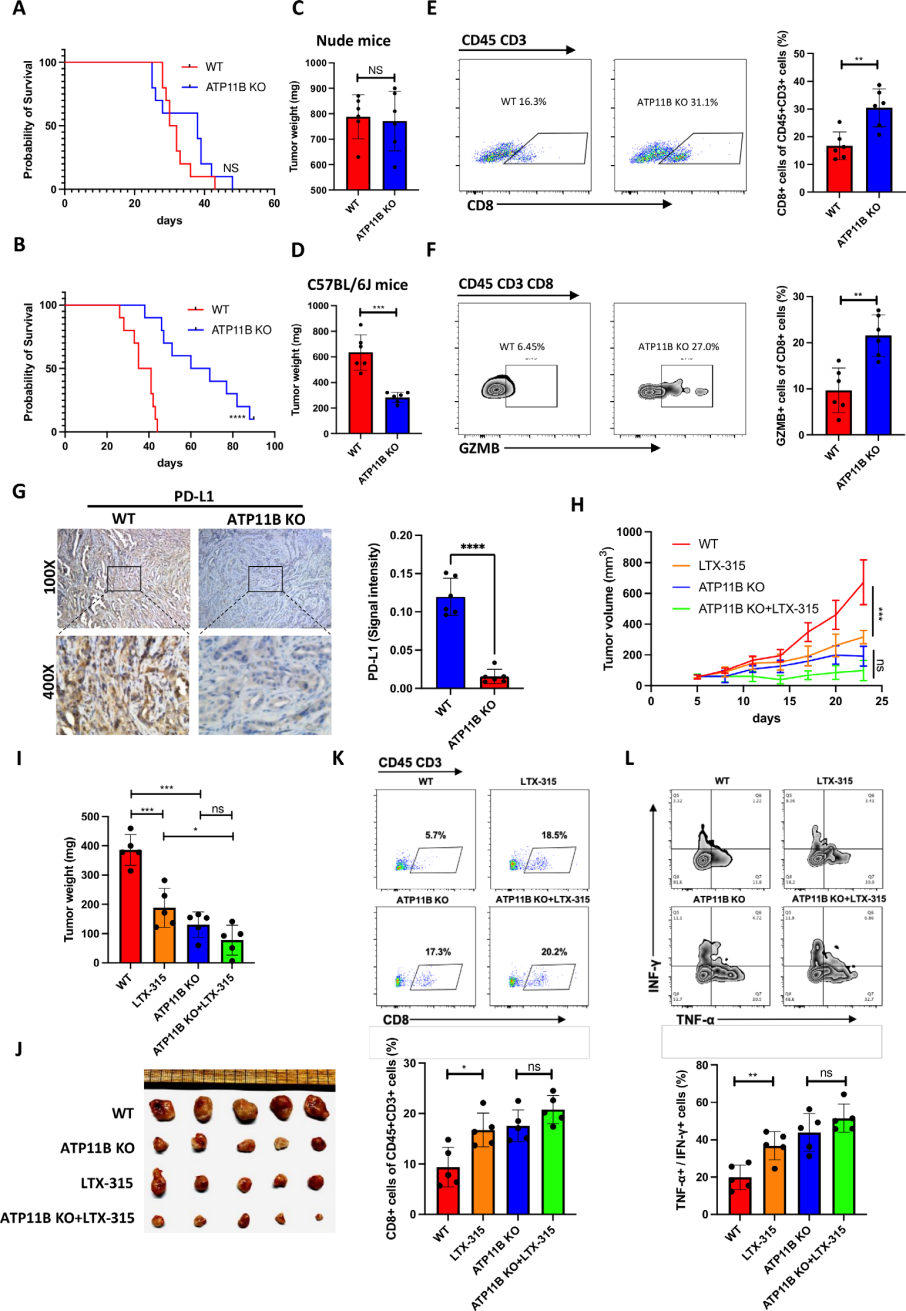
ATP11B depletion activates anti-pancreatic cancer immunity. (A–F) Inhibition of pancreatic tumor growth by ATP11B depletion in an immune system-dependent manner. (A–B) WT and ATP11B KO KPC cells separately orthotopically inoculated into the immunodeficient and immunocompetent mice. Survival curves of immunodeficient (A) and immunocompetent mice (B). WT and ATP11B KO KPC cells were separately orthotopically inoculated into the immunodeficient and immunocompetent mice (n=6). Representative images and weight of tumors in the immunodeficient (C) and immunocompetent mice (D) were individually recorded at the experimental endpoints. Representative FACS images of tumor-infiltrating lymphocytes in immunocompetent mice were shown and further quantified (E–F). (G) Representative images of PD-L1 immunohistochemical staining shown and further quantified. (H–L) Abolition of the therapeutic efficacy of LTX-315 in pancreatic cancer by ATP11B KO. WT and ATP11B KO KPC cells subcutaneous inoculated into the immunocompetent mice (n=5) treated with LTX-315. Growth curves of tumors were recorded at the indicated time points (H). Representative images of tumor weight (I) and tumors (J) were individually recorded at the experimental endpoints. Representative images of FACS analysis of T cell infiltration (K) and T cell function (L) were individually shown and further quantified. Results are presented as mean±SD from one representative experiment. *p<0.05, **p<0.01, ***p<0.001 by a two-tailed t-test; KO, knockout; NS, not significant; PD-L1, programmed cell death ligand 1; WT, wild type.

The potential impact of targeting ATP11B on the basic characteristics of tumor growth was evaluated in vitro to explore the effect of this potential therapy against pancreatic cancer. No significant stimulation or inhibitory effect was observed on cell colony formation (online supplemental figure S12A–D) and proliferation (online supplemental figure S12E, F) in pancreatic cancer cell lines neither with the overexpression of ATP11B nor with its depletion. In contrast, ATP11B depletion in the mouse models significantly prolonged the survival of C57BL/6 mice, while it did not affect the prognosis of nude mice (figure 8A, B). Consistently, no inhibitory effect on tumor growth was observed in the immunodeficient nude mice after the depletion of ATP11B (figure 8C). In contrast, ATP11B depletion largely suppressed tumor growth in immunocompetent C57BL/6 mice (figure 8D). Moreover, ATP11B depletion resulted in an increase of the infiltration (figure 8E) and activation (figure 8F) of CD8 +T cells. As demonstrated by IHC staining, significantly reduced PD-L1 expression was also observed in tumor cells after ATP11B depletion (figure 8G), suggesting the strong immune-regulatory function of ATP11B in pancreatic cancer. KPC tumors with or without ATP11B depletion were treated with LTX-315 to investigate whether LTX-315 induced an antitumor immunity in an ATP11B-dependent way. Interestingly, LTX-315-induced tumor growth inhibition was largely abolished by ATP11B depletion (figure 8H–J). Furthermore, LTX-315 significantly increased CD8 +T infiltration and activation of CD8 +T cells in WT tumors, while no difference of immune infiltration was observed in ATP11B KO tumors (figure 8K, L). Furthermore, the virtual screening approach was used to identify 3D protein structure as well as the active site and small molecule binding pocket of ATP11B, useful in the development of targeted drugs in the future (online supplemental figure S13A–C). Collectively, these findings revealed the critical role of ATP11B in the immune resistance of pancreatic cancer, indicating the targeting potential of ATP11B in pancreatic cancer immunotherapy (online supplemental figure S14A).

## Discussion

Several studies have confirmed intratumoral administration of LTX-315 as a promising treatment strategy in the immune-oncology field because of its ability to induce tumor cell lysis and subsequently expose tumor antigens and DAMPs to immune systems. However, only the oncolytic effects are not enough to fully explain the strong efficacy of LTX-315 in boosting antitumor immunity, compared with numerous cell lysis agents. Therefore, there is still an urgent need to investigate the underlying mechanism used by LTX-315 to induce antitumor immunity. The current study revealed that LTX-315 targeted ATP11B, a type 4 P-type ATPase (P4-ATPase) localized in the early/recycling endosomes, which was a critical regulator in the maintenance of PD-L1 expression in pancreatic cancer. To our knowledge, this is the first report revealing the immune-suppressive effect of ATP11B. Previous studies showed that transmembrane ATPases import a variety of metabolites critical for cell metabolism, export waste that can compromise cellular processes, and are crucial in the regulation of cellular vesicle trafficking and membrane proteins.^38 39^ These molecules play significant roles in mediating an immune suppressive microenvironment in the tumor and ATPases are identified as promising targets for immunotherapy and combination therapy to combat tumors.^40 41^ Kulshrestha *et al* showed that V-ATPase inhibition using a monoclonal antibody enhances the antitumor immune response, drastically inhibiting ovarian tumor growth without evident side effects.^40^ Our previous study demonstrated that MET interacts with V-ATPase and suppresses liver cancer immunogenicity through the activation of the mammalian target of rapamycin.^41^ Moreover, the V-ATPase inhibitor Con A significantly enhances the efficacy of chemotherapy-based vaccination in a mouse model. However, the detailed mechanism of action of ATPases in tumor immune escape is still largely unknown.

In this study, we found that LTX-315 treatment inhibited PD-L1 expression by targeting the ATP11B/CMTM6 axis. A variety of molecules have been reported involved in regulating PD-L1 stability, including CSN5, CMTM6, CDK4, USP22, and GSK3β.^37 42–45^ Since PD-L1 degradation is promoted by drugs targeting the above-mentioned factors, including etoposide, metformin, and gefitinib, the combination therapy involving such drugs and anti-PD-L1 or anti-CTLA4 was developed to maximize the clinical benefit of immune checkpoints.^46–48^ The current study demonstrated that the depletion of ATP11B downregulated CMTM6, consequently resulting in PD-L1 degradation by lysosomes. CMTM6 is widely expressed on the plasma membrane of various tumor cells and induces PD-L1 post-translational modification by binding it on the plasma membrane as well as recycling endosomes to remain expressed in the cell surface through the protection against lysosomal-mediated degradation.^37^ Depletion of CMTM6 in tumor cells reduces the half-life of PD-L1 protein and relieves T-cell immunosuppression. In addition, the clinical significance of CMTM6 is further confirmed in head and neck squamous cell carcinoma, glioma, and gastric cancer.^49–51^ CMTM6 expression refines the prognostic value of PD-L1 in pancreatic cancer, and PD-L1^high^ CMTM6^high^ is associated with the worst prognosis, with a 27% 2-year overall survival.^52^ Although these results underline the therapeutic potential of CMTM6 as a promising target in combination with PD-1/PD-L1 blockade therapy, the targetability of CMTM6 is still largely unknown, indicating that the regulatory mechanism itself needs to be further investigated. Moreover, the development of small molecular inhibitors against CMTM6 is difficult because of its role as an adaptor physically interacting with other molecules and because of its migration between endosome and cell plasma. Therefore, ATP11B might be a better target than CMTM6 in the potential development of targeted drugs for immunotherapy and combination therapy due to its enzymatic nature.

It is noteworthy that ATP11B expression is associated with a high tumor grade and plays a critical role in cisplatin resistance in ovarian cancer.^53^ Moreover, it is colocalized with fluorescent cisplatin and vesicular trafficking proteins, suggesting its ability to promote the export of cisplatin from tumor cells using a secretory vesicular transporting system.^53^ ATP11B depletion restores the tumor sensitivity to chemotherapy and the combination of cisplatin and ATP11B depletion therapy leads to significant inhibition of tumor growth in a mouse model.^53^ Of note, our study also revealed the close correlation between the pathologic grade and ATP11B expression. In addition, ATP11B is significantly upregulated in PDAC and lung squamous cell carcinoma and its high expression is associated with a lower survival rate in patients with adrenocortical carcinoma, acute myeloid leukemia, brain lower grade glioma, lung adenocarcinoma, and PDAC. CMTM6 is also identified as a prognostic biomarker and a critical regulator of PD-L1 in many tumors, including breast cancer, gastric cancer, non-small cell lung cancer, and hepatocellular carcinoma, and our results from TCGA data sets are consistent with this result in these studies.^49–51 54 55^ Notably, while the survival curves of ATP11B^high^ group and ATP11B^low^ group or CMTM6^high^ group and CMTM6^low^ group were similar in patients with ‘cold’ tumor, both ATP11B^high^ group and CMTM6^high^ group were correlated with a remarkable lower survival rate in patients with ‘hot’ tumor, suggesting that both ATP11B and CMTM6 suppressed the antitumor immunity through the inhibition of the CD8 +T cell priming. Collectively, these results demonstrated that the ATP11B-CMTM6-PD-L1 axis might be universally activated and could rearrange the tumor immune microenvironment in multiple cancer types. Therefore, the development of drugs targeting ATP11B-CMTM6-PD-L1 might be beneficial for a wide spectrum of patients with different types of malignancies.

It should be also noted that, in addition to ATP11B, we found LTX-315 could target many other molecules which might be also involved in the expression regulation of PD-L1, even other immune checkpoints. Moreover, in our study, LTX-315 treatment also resulted in a significant alteration of multiple gene pathways including the JAK-STAT pathway, IL-6 pathway, and pathways associated with cytokine production. The activation of the JAK/STAT pathway contributes to IFN-mediated PD-L1 upregulation in several types of cancers.^56–58^ The activation of the IL-6/JAK1 pathway leads to PD-L1 Y112 phosphorylation and maintains PD-L1 stability.^59^ Cytokines including IFN-γ, TNF-α, IL-1α, and IL-27 are also able to induce PD-L1 expression in tumor cells.^56 58 60^ Since the alteration of these pathways might also contribute to the influence of LTX-315 on PD-L1 and it-mediated immune resistance, further studies on the context-dependent action mechanisms of LTX-315 in cancer treatment are still needed.

10.1136/jitc-2021-004129.supp2 Supplementary data

## Data Availability

Data are available upon reasonable request. The data sets used and/or analyzed during the current study are available from the corresponding authors upon reasonable request.

## References

[1] Siegel RL, Miller KD, Fuchs HE, et al. Cancer statistics, 2022. CA Cancer J Clin 2022;72:7–33. 10.3322/caac.21708 35020204

[2] Makohon-Moore A, Iacobuzio-Donahue CA. Pancreatic cancer biology and genetics from an evolutionary perspective. Nat Rev Cancer 2016;16:553–65. 10.1038/nrc.2016.66 27444064 PMC5739515

[3] Huang X, Zhang G, Tang T, et al. Identification of tumor antigens and immune subtypes of pancreatic adenocarcinoma for mRNA vaccine development. Mol Cancer 2021;20:44. 10.1186/s12943-021-01310-0 33648511 PMC7917175

[4] Strobel O, Neoptolemos J, Jäger D, et al. Optimizing the outcomes of pancreatic cancer surgery. Nat Rev Clin Oncol 2019;16:11–26. 10.1038/s41571-018-0112-1 30341417

[5] Nevala-Plagemann C, Hidalgo M, Garrido-Laguna I. From state-of-the-art treatments to novel therapies for advanced-stage pancreatic cancer. Nat Rev Clin Oncol 2020;17:108–23. 10.1038/s41571-019-0281-6 31705130

[6] Mizrahi JD, Surana R, Valle JW, et al. Pancreatic cancer. Lancet 2020;395:2008–20. 10.1016/S0140-6736(20)30974-0 32593337

[7] Bockhorn M, Uzunoglu FG, Adham M, et al. Borderline resectable pancreatic cancer: a consensus statement by the International Study group of pancreatic surgery (ISGPS). Surgery 2014;155:977–88. 10.1016/j.surg.2014.02.001 24856119

[8] Suker M, Beumer BR, Sadot E, et al. Folfirinox for locally advanced pancreatic cancer: a systematic review and patient-level meta-analysis. Lancet Oncol 2016;17:801–10. 10.1016/S1470-2045(16)00172-8 27160474 PMC5527756

[9] Gresham GK, Wells GA, Gill S, et al. Chemotherapy regimens for advanced pancreatic cancer: a systematic review and network meta-analysis. BMC Cancer 2014;14:471. 10.1186/1471-2407-14-471 24972449 PMC4097092

[10] Topalian SL. Targeting immune checkpoints in cancer therapy. JAMA 2017;318:1647–8. 10.1001/jama.2017.14155 28885639

[11] Chen L, Han X. Anti–PD-1/PD-L1 therapy of human cancer: past, present, and future. J Clin Invest 2015;125:3384–91. 10.1172/JCI80011 26325035 PMC4588282

[12] Balachandran VP, Beatty GL, Dougan SK. Broadening the impact of immunotherapy to pancreatic cancer: challenges and opportunities. Gastroenterology 2019;156:2056–72. 10.1053/j.gastro.2018.12.038 30660727 PMC6486864

[13] Neoptolemos JP, Kleeff J, Michl P, et al. Therapeutic developments in pancreatic cancer: current and future perspectives. Nat Rev Gastroenterol Hepatol 2018;15:333–48. 10.1038/s41575-018-0005-x 29717230

[14] Bear AS, Vonderheide RH, O'Hara MH. Challenges and opportunities for pancreatic cancer immunotherapy. Cancer Cell 2020;38:788–802. 10.1016/j.ccell.2020.08.004 32946773 PMC7738380

[15] Li E, Huang X, Zhang G, et al. Combinational blockade of Met and PD-L1 improves pancreatic cancer immunotherapeutic efficacy. J Exp Clin Cancer Res 2021;40:279. 10.1186/s13046-021-02055-w 34479614 PMC8414725

[16] Zhang X, Huang X, Xu J, et al. Nek2 inhibition triggers anti-pancreatic cancer immunity by targeting PD-L1. Nat Commun 2021;12:4536. 10.1038/s41467-021-24769-3 34315872 PMC8316469

[17] Koikawa K, Kibe S, Suizu F, et al. Targeting Pin1 renders pancreatic cancer eradicable by synergizing with immunochemotherapy. Cell 2021;184:e27:4753–71. 10.1016/j.cell.2021.07.020 PMC8557351 34388391

[18] Henriksen A, Dyhl-Polk A, Chen I, et al. Checkpoint inhibitors in pancreatic cancer. Cancer Treat Rev 2019;78:17–30. 10.1016/j.ctrv.2019.06.005 31325788

[19] Huang X, Tang T, Wang X, et al. Calreticulin couples with immune checkpoints in pancreatic cancer. Clin Transl Med 2020;10:36–44. 10.1002/ctm2.10 32508042 PMC7239268

[20] Tang T, Huang X, Zhang G, et al. Advantages of targeting the tumor immune microenvironment over blocking immune checkpoint in cancer immunotherapy. Sig Transduct Target Ther 2021;6:72. 10.1038/s41392-020-00449-4 PMC7896069 33608497

[21] Camilio KA, Rekdal O, Sveinbjörnsson B. LTX-315 (Oncopore™): a short synthetic anticancer peptide and novel immunotherapeutic agent. Oncoimmunology 2014;3:e29181. 10.4161/onci.29181 25083333 PMC4108458

[22] Vitale I, Yamazaki T, Wennerberg E, et al. Targeting cancer heterogeneity with immune responses driven by oncolytic peptides. Trends Cancer 2021;7:557–72. 10.1016/j.trecan.2020.12.012 33446447

[23] Sveinbjørnsson B, Camilio KA, Haug BE, et al. LTX-315: a first-in-class oncolytic peptide that reprograms the tumor microenvironment. Future Med Chem 2017;9:1339–44. 10.4155/fmc-2017-0088 28490192

[24] Liao H-W, Garris C, Pfirschke C, et al. LTX-315 sequentially promotes lymphocyte-independent and lymphocyte-dependent antitumor effects. Cell Stress 2019;3:348–60. 10.15698/cst2019.11.204 31799501 PMC6859426

[25] Yamazaki T, Wennerberg E, Hensler M, et al. LTX-315-enabled, radiotherapy-boosted immunotherapeutic control of breast cancer by NK cells. Oncoimmunology 2021;10:1962592. 10.1080/2162402X.2021.1962592 34408925 PMC8366543

[26] Camilio KA, Wang M-Y, Mauseth B, et al. Combining the oncolytic peptide LTX-315 with doxorubicin demonstrates therapeutic potential in a triple-negative breast cancer model. Breast Cancer Res 2019;21:9. 10.1186/s13058-018-1092-x 30670061 PMC6343247

[27] Forveille S, Zhou H, Sauvat A, et al. The oncolytic peptide LTX-315 triggers necrotic cell death. Cell Cycle 2015;14:3506–12. 10.1080/15384101.2015.1093710 26566869 PMC4825625

[28] Nestvold J, Wang M-Y, Camilio KA, et al. Oncolytic peptide LTX-315 induces an immune-mediated abscopal effect in a rat sarcoma model. Oncoimmunology 2017;6:e1338236. 10.1080/2162402X.2017.1338236 28920000 PMC5593701

[29] Spicer J, Marabelle A, Baurain J-F, et al. Safety, antitumor activity, and T-cell responses in a dose-ranging phase I trial of the oncolytic peptide LTX-315 in patients with solid tumors. Clin Cancer Res 2021;27:2755–63. 10.1158/1078-0432.CCR-20-3435 33542073

[30] Xia Y, Wei J, Zhao S, et al. Systemic administration of polymersomal oncolytic peptide LTX-315 combining with CpG adjuvant and anti-PD-1 antibody boosts immunotherapy of melanoma. J Control Release 2021;336:262–73. 10.1016/j.jconrel.2021.06.032 34174350

[31] Ru B, Wong CN, Tong Y, et al. TISIDB: an integrated Repository portal for tumor-immune system interactions. Bioinformatics 2019;35:4200–2. 10.1093/bioinformatics/btz210 30903160

[32] Charoentong P, Finotello F, Angelova M, et al. Pan-Cancer Immunogenomic analyses reveal Genotype-Immunophenotype relationships and predictors of response to checkpoint blockade. Cell Rep 2017;18:248–62. 10.1016/j.celrep.2016.12.019 28052254

[33] Tang Z, Kang B, Li C, et al. GEPIA2: an enhanced web server for large-scale expression profiling and interactive analysis. Nucleic Acids Res 2019;47:W556–60. 10.1093/nar/gkz430 31114875 PMC6602440

[34] Newman AM, Liu CL, Green MR, et al. Robust enumeration of cell subsets from tissue expression profiles. Nat Methods 2015;12:453–7. 10.1038/nmeth.3337 25822800 PMC4739640

[35] Yoshihara K, Shahmoradgoli M, Martínez E, et al. Inferring tumour purity and stromal and immune cell admixture from expression data. Nat Commun 2013;4:2612. 10.1038/ncomms3612 24113773 PMC3826632

[36] Mezzadra R, Sun C, Jae LT, et al. Identification of CMTM6 and CMTM4 as PD-L1 protein regulators. Nature 2017;549:106–10. 10.1038/nature23669 28813410 PMC6333292

[37] Burr ML, Sparbier CE, Chan Y-C, et al. CMTM6 maintains the expression of PD-L1 and regulates anti-tumour immunity. Nature 2017;549:101–5. 10.1038/nature23643 28813417 PMC5706633

[38] Lyons JA, Timcenko M, Dieudonné T, et al. P4-Atpases: how an old dog learnt new tricks — structure and mechanism of lipid flippases. Curr Opin Struct Biol 2020;63:65–73. 10.1016/j.sbi.2020.04.001 32492637

[39] Tone T, Nakayama K, Takatsu H, et al. Atpase reaction cycle of P4‐ATPases affects their transport from the endoplasmic reticulum. FEBS Lett 2020;594:412–23. 10.1002/1873-3468.13629 31571211

[40] Kulshrestha A, Katara GK, Ibrahim SA, et al. In vivo anti-V-ATPase antibody treatment delays ovarian tumor growth by increasing antitumor immune responses. Mol Oncol 2020;14:2436–54. 10.1002/1878-0261.12782 32797726 PMC7530789

[41] Huang X, Xu X, Wang X, et al. The Akt-independent MET-V-ATPase-MTOR axis suppresses liver cancer vaccination. Signal Transduct Target Ther 2020;5:122. 10.1038/s41392-020-0179-x 32764535 PMC7414041

[42] Lim S-O, Li C-W, Xia W, et al. Deubiquitination and stabilization of PD-L1 by CSN5. Cancer Cell 2016;30:925–39. 10.1016/j.ccell.2016.10.010 27866850 PMC5171205

[43] Zhang J, Bu X, Wang H, et al. Cyclin D-Cdk4 kinase destabilizes PD-L1 via cullin 3-SPOP to control cancer immune surveillance. Nature 2018;553:91–5. 10.1038/nature25015 29160310 PMC5754234

[44] Li C-W, Lim S-O, Xia W, et al. Glycosylation and stabilization of programmed death ligand-1 suppresses T-cell activity. Nat Commun 2016;7:12632. 10.1038/ncomms12632 27572267 PMC5013604

[45] Huang X, Zhang Q, Lou Y, et al. Usp22 deubiquitinates CD274 to suppress anticancer immunity. Cancer Immunol Res 2019;7:1580–90. 10.1158/2326-6066.CIR-18-0910 31399419

[46] Hsu JM, Xia W, Hsu YH. STT3-dependent PD-L1 accumulation on cancer stem cells promotes immune evasion. Nat Commun 20181908;9. 10.1038/s41467-018-04313-6 PMC5954021 29765039

[47] Cha J-H, Yang W-H, Xia W, et al. Metformin promotes antitumor immunity via endoplasmic-reticulum-associated degradation of PD-L1. Mol Cell 2018;71:606–20. 10.1016/j.molcel.2018.07.030 30118680 PMC6786495

[48] Miller KD, Siegel RL, Lin CC, et al. Cancer treatment and survivorship statistics, 2016. CA Cancer J Clin 2016;66:271–89. 10.3322/caac.21349 27253694

[49] Chen L, Yang Q-C, Li Y-C, et al. Targeting CMTM6 suppresses stem cell-like properties and enhances antitumor immunity in head and neck squamous cell carcinoma. Cancer Immunol Res 2020;8:179–91. 10.1158/2326-6066.CIR-19-0394 31771985

[50] Guan X, Zhang C, Zhao J, et al. CMTM6 overexpression is associated with molecular and clinical characteristics of malignancy and predicts poor prognosis in gliomas. EBioMedicine 2018;35:233–43. 10.1016/j.ebiom.2018.08.012 30131308 PMC6156716

[51] Ubukata Y, Ogata K, Sohda M, et al. Role of PD-L1 expression during the progression of submucosal gastric cancer. Oncology 2021;99:15–22. 10.1159/000509033 33113541

[52] Mamessier E, Birnbaum DJ, Finetti P, et al. CMTM6 stabilizes PD-L1 expression and refines its prognostic value in tumors. Ann Transl Med 2018;6:54. 10.21037/atm.2017.11.26 29610746 PMC5879522

[53] Moreno-Smith M, Halder JB, Meltzer PS, et al. ATP11B mediates platinum resistance in ovarian cancer. J Clin Invest 2013;123:2119–30. 10.1172/JCI65425 23585472 PMC3635722

[54] Zugazagoitia J, Liu Y, Toki M, et al. Quantitative assessment of CMTM6 in the tumor microenvironment and association with response to PD-1 pathway blockade in advanced-stage Non–Small cell lung cancer. J Thorac Oncol 2019;14:2084–96. 10.1016/j.jtho.2019.09.014 31605795 PMC6951804

[55] Liu L-L, Zhang S-W, Chao X, et al. Coexpression of CMTM6 and PD-L1 as a predictor of poor prognosis in macrotrabecular-massive hepatocellular carcinoma. Cancer Immunol Immunother 2021;70:417-429. 10.1007/s00262-020-02691-9 32770259 PMC7889680

[56] Lamberti G, Sisi M, Andrini E, et al. The mechanisms of PD-L1 regulation in non-small-cell lung cancer (NSCLC): which are the involved players? Cancers 2020;12. 10.3390/cancers12113129. [Epub ahead of print: 26 10 2020]. 10.3390/cancers12113129 PMC7692442 33114576

[57] Miao D, Margolis CA, Gao W, et al. Genomic correlates of response to immune checkpoint therapies in clear cell renal cell carcinoma. Science 2018;359:801–6. 10.1126/science.aan5951 29301960 PMC6035749

[58] Zhou L, Zhang Y, Wang Y, et al. A dual role of type I interferons in antitumor immunity. Adv Biosyst 2020;4:e1900237:1900237. 10.1002/adbi.201900237 33245214

[59] Chan L-C, Li C-W, Xia W, et al. IL-6/JAK1 pathway drives PD-L1 Y112 phosphorylation to promote cancer immune evasion. J Clin Invest 2019;129:3324–38. 10.1172/JCI126022 31305264 PMC6668668

[60] Antonangeli F, Natalini A, Garassino MC, et al. Regulation of PD-L1 expression by NF-κB in cancer. Front Immunol 2020;11:584626. 10.3389/fimmu.2020.584626 33324403 PMC7724774

