# Supplementary material for: Oncolytic peptide LTX-315 induces anti-pancreatic cancer immunity by targeting the ATP11B-PD-L1 axis
Source: J Immunother Cancer. 2022 Mar 14;10(3):e004129. doi: 10.1136/jitc-2021-004129 (PMC8921947; doi:10.1136/jitc-2021-004129)
Supplement: Supplementary data [file jitc-2021-004129supp001.pdf]

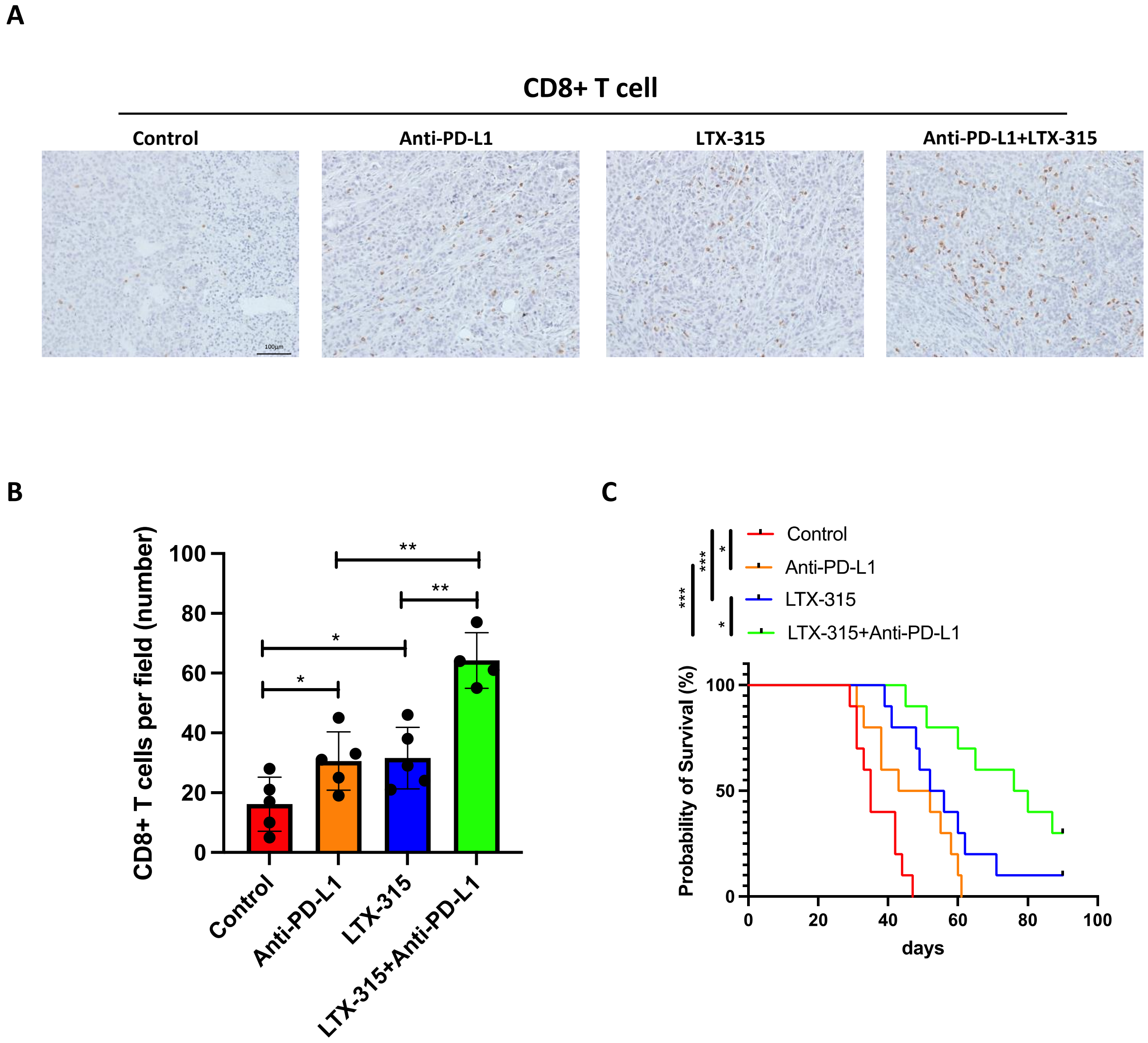
A
CD8+ T cell
Control
Anti-PD-L1
LTX-315
Anti-PD-L1+LTX-315
B
CD8+ T cells per field (number)
100
80
60
40
20
0
**
*
Control
Anti-PD-L1
LTX-315
LTX-315+Anti-PD-L1
C
Control
Anti-PD-L1
LTX-315
LTX-315+Anti-PD-L1
***
*
Probability of Survival (%)
100
50
0
0
20
40
60
80
100
days

Figure S1

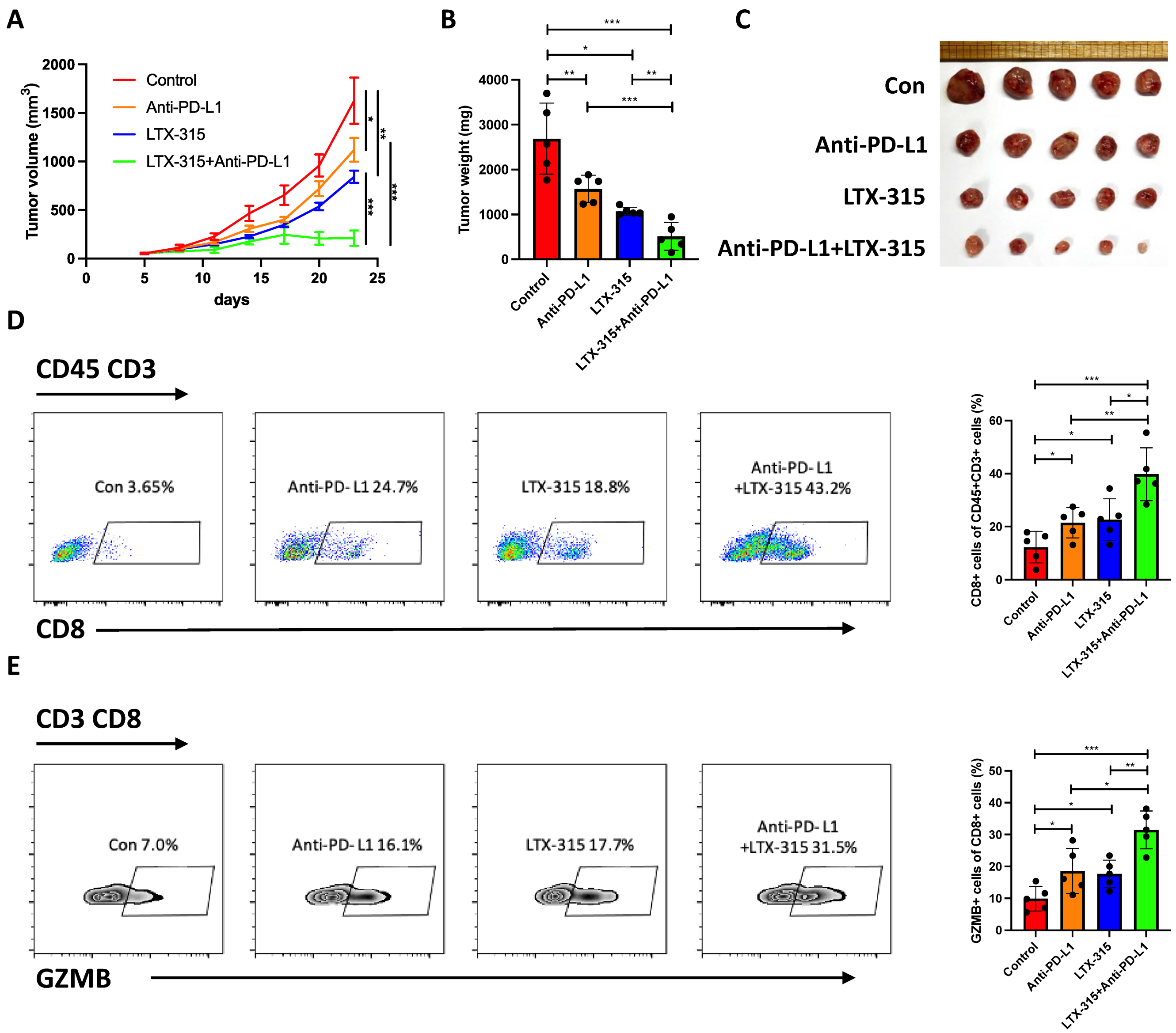
A
B
C
D
E
Control
Anti-PD-L1
LTX-315
LTX-315+Anti-PD-L1
Tumor volume (mm3)
days
Tumor weight (mg)
Con
Anti-PD-L1
LTX-315
Anti-PD-L1+LTX-315
CD45 CD3
Con 3.65%
Anti-PD- L1 24.7%
LTX-315 18.8%
Anti-PD- L1 +LTX-315 43.2%
CD8
CD8+ cells of CD45+CD3+ cells (%)
CD3 CD8
Con 7.0%
Anti-PD- L1 16.1%
LTX-315 17.7%
Anti-PD- L1 +LTX-315 31.5%
GZMB
GZMB+ cells of CD8+ cells (%)

Figure S2

A

Group	Sample	B cells naive	B cells memory	Plasma cells	T cells CD8	T cells CD4 naive	T cells CD4 memory resting	T cells CD4 memory activated	T cells follicular helper	T cells regulatory (Tregs)	T cells gamma delta	NK cells resting	NK cells activated
Control	6	0	0.075	0.017	0.049	0	0	0	0.04	0.006	0	0.038	0
Control	5	0	0.072	0.023	0.041	0	0	0.011	0.038	0.019	0	0.042	0
Control	4	0	0.046	0.012	0.034	0	0.003	0.01	0.03	0.009	0	0.034	0
Control	3	0	0.076	0.015	0.079	0	0	0.023	0.033	0.009	0	0.054	0
Control	2	0	0.083	0.011	0.037	0	0.008	0.003	0.039	0.002	0	0.064	0
Control	1	0	0.049	0.028	0.08	0	0	0.023	0.045	0.015	0	0.046	0
LTX-315	6	0	0.033	0.048	0.124	0	0	0.075	0.023	0.011	0	0.058	0.018
LTX-315	5	0.01	0.02	0.021	0.139	0	0	0.091	0.021	0.011	0	0.071	0.014
LTX-315	4	0	0.055	0.013	0.094	0	0	0.086	0.029	0.013	0	0.08	0
LTX-315	3	0	0.055	0.025	0.105	0	0	0.062	0.041	0.007	0	0.077	0
LTX-315	2	0	0.039	0.023	0.098	0	0	0.081	0.023	0.001	0	0.046	0.015
LTX-315	1	0	0.049	0.018	0.078	0	0	0.067	0.025	0.006	0	0.089	0.001

Group	Sample	Monocytes	Macrophages M0	Macrophages M1	Macrophages M2	Dendritic cells resting	Dendritic cells activated	Mast cells resting	Mast cells activated	Eosinophils	Neutrophils	P-value	Correlation	RMSE
Control	6	0	0.319	0.065	0.081	0.237	0	0	0.071	0.002	0	0.000	0.823	0.633
Control	5	0	0.289	0.048	0.053	0.247	0	0	0.079	0.032	0.005	0.000	0.813	0.655
Control	4	0	0.389	0.056	0.04	0.185	0	0	0.095	0.047	0.011	0.000	0.801	0.636
Control	3	0	0.297	0.047	0.076	0.2	0.006	0.001	0.049	0.034	0	0.000	0.781	0.681
Control	2	0	0.335	0.075	0.044	0.188	0	0	0.085	0.026	0	0.000	0.791	0.660
Control	1	0.015	0.234	0.048	0.049	0.267	0.013	0	0.062	0.027	0	0.000	0.810	0.675
LTX-315	6	0.059	0.168	0.06	0.047	0.236	0	0.023	0.018	0	0	0.000	0.809	0.700
LTX-315	5	0.03	0.175	0.05	0.101	0.141	0.011	0	0.078	0.015	0	0.000	0.782	0.702
LTX-315	4	0	0.3	0.058	0.066	0.131	0	0	0.073	0.002	0	0.000	0.804	0.663
LTX-315	3	0.005	0.305	0.06	0.07	0.135	0.003	0	0.05	0	0	0.000	0.807	0.662
LTX-315	2	0	0.279	0.05	0.067	0.21	0	0	0.043	0.021	0.003	0.000	0.841	0.640
LTX-315	1	0.019	0.281	0.054	0.052	0.173	0.003	0	0.08	0	0.004	0.000	0.806	0.665

Figure S3

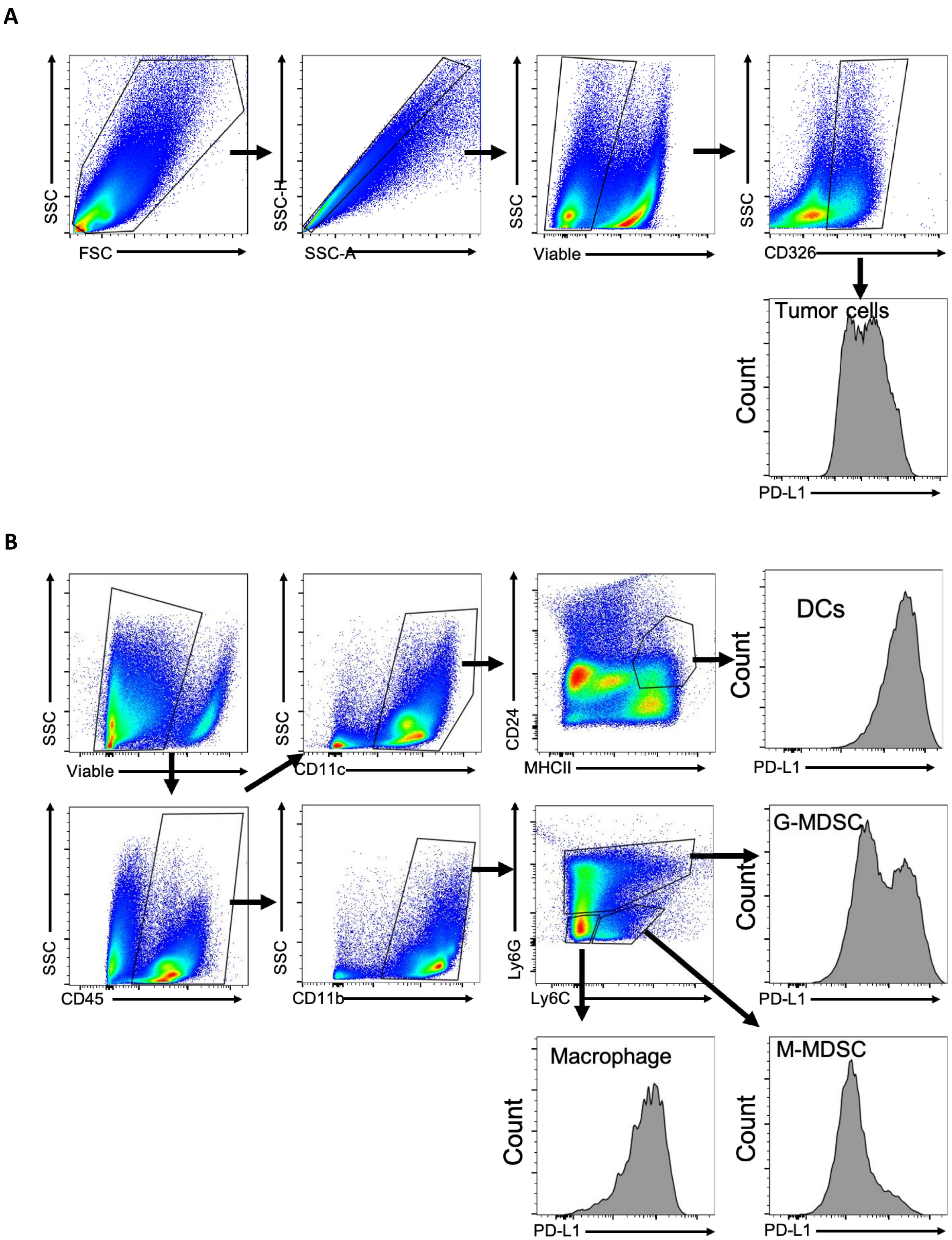
A
SSC
FSC
SSC-H
SSC-A
SSC
Viable
SSC
CD326
Tumor cells
Count
PD-L1
B
SSC
Viable
SSC
CD11c
CD24
MHCII
DCs
Count
PD-L1
SSC
CD45
SSC
CD11b
Ly6G
Ly6C
G-MDSC
Count
PD-L1
Macrophage
Count
PD-L1
M-MDSC
Count
PD-L1

Figure S4

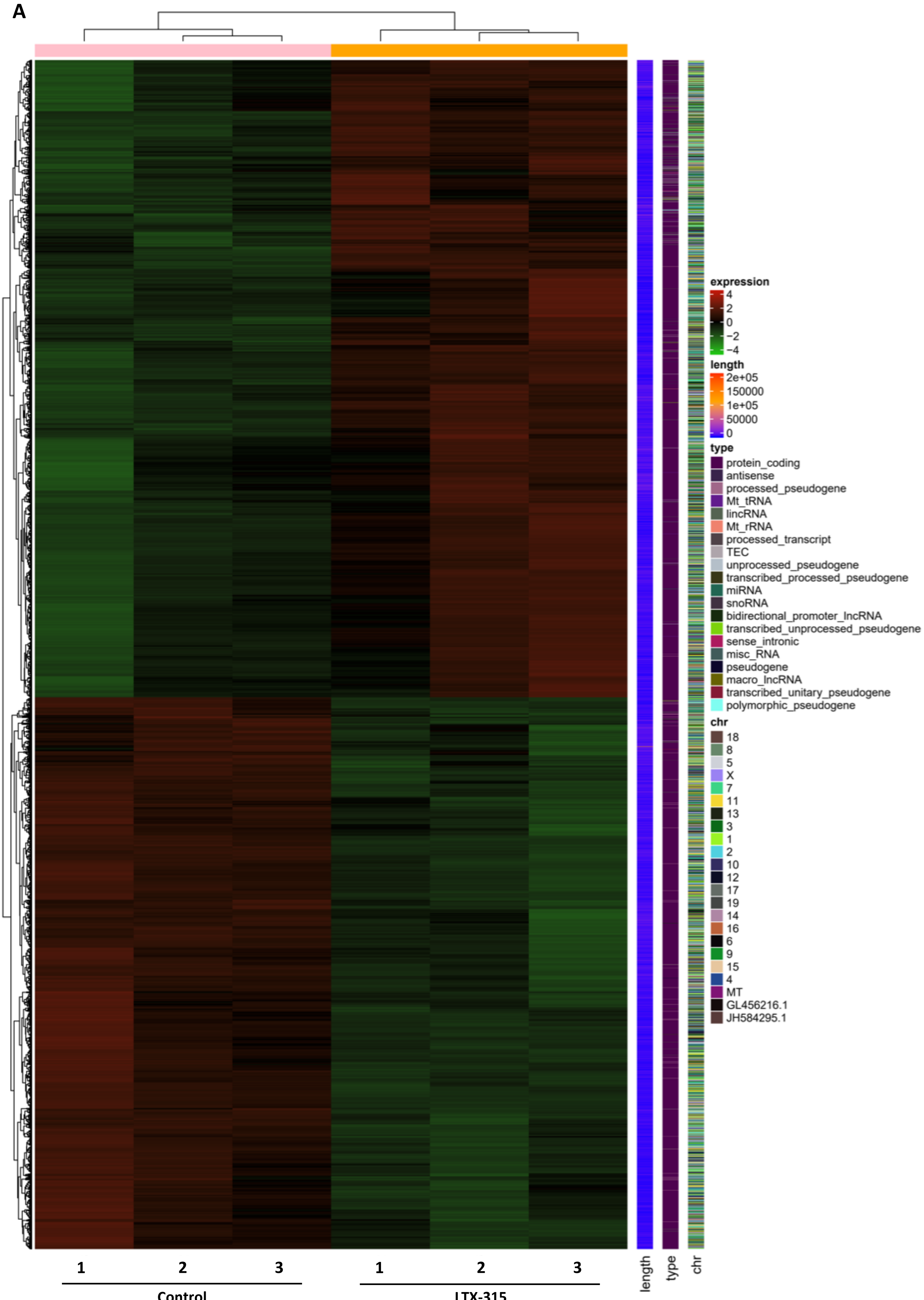
A
expression
4
2
0
−2
−4
length
2e+05
150000
1e+05
50000
0
type
protein_coding
antisense
processed_pseudogene
Mt_tRNA
lincRNA
Mt_rRNA
processed_transcript
TEC
unprocessed_pseudogene
transcribed_processed_pseudogene
miRNA
snoRNA
bidirectional_promoter_lncRNA
transcribed_unprocessed_pseudogene
sense_intronic
misc_RNA
pseudogene
macro_lncRNA
transcribed_unitary_pseudogene
polymorphic_pseudogene
chr
18
8
5
X
7
11
13
3
1
2
10
12
17
19
14
16
6
9
15
4
MT
GL456216.1
JH584295.1
1
2
3
1
2
3
Control
LTX-315
length
type
chr

Figure S5

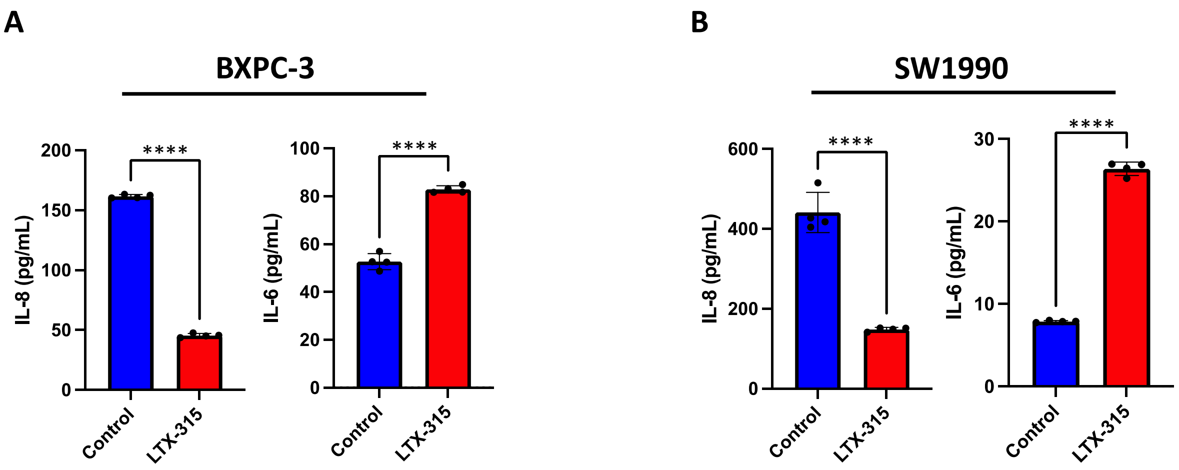
A
BXPC-3
B
SW1990
IL-8 (pg/mL)
IL-6 (pg/mL)
****
Control
LTX-315
200
150
100
50
0
80
60
40
20
600
400
30
10

Figure S6

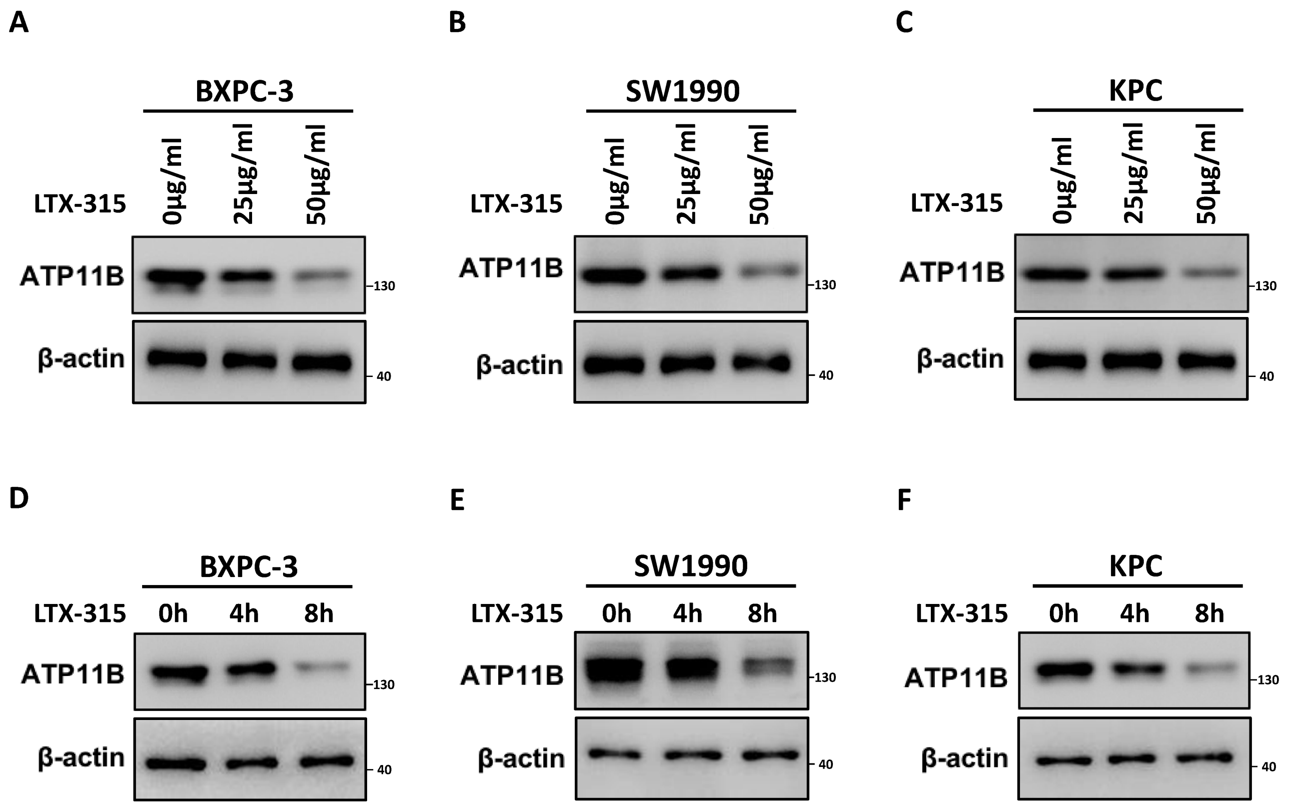
A
B
C
BXPC-3
SW1990
KPC
LTX-315
0μg/ml
25μg/ml
50μg/ml
ATP11B
β-actin
130
40
D
E
F
0h
4h
8h

Figure S7

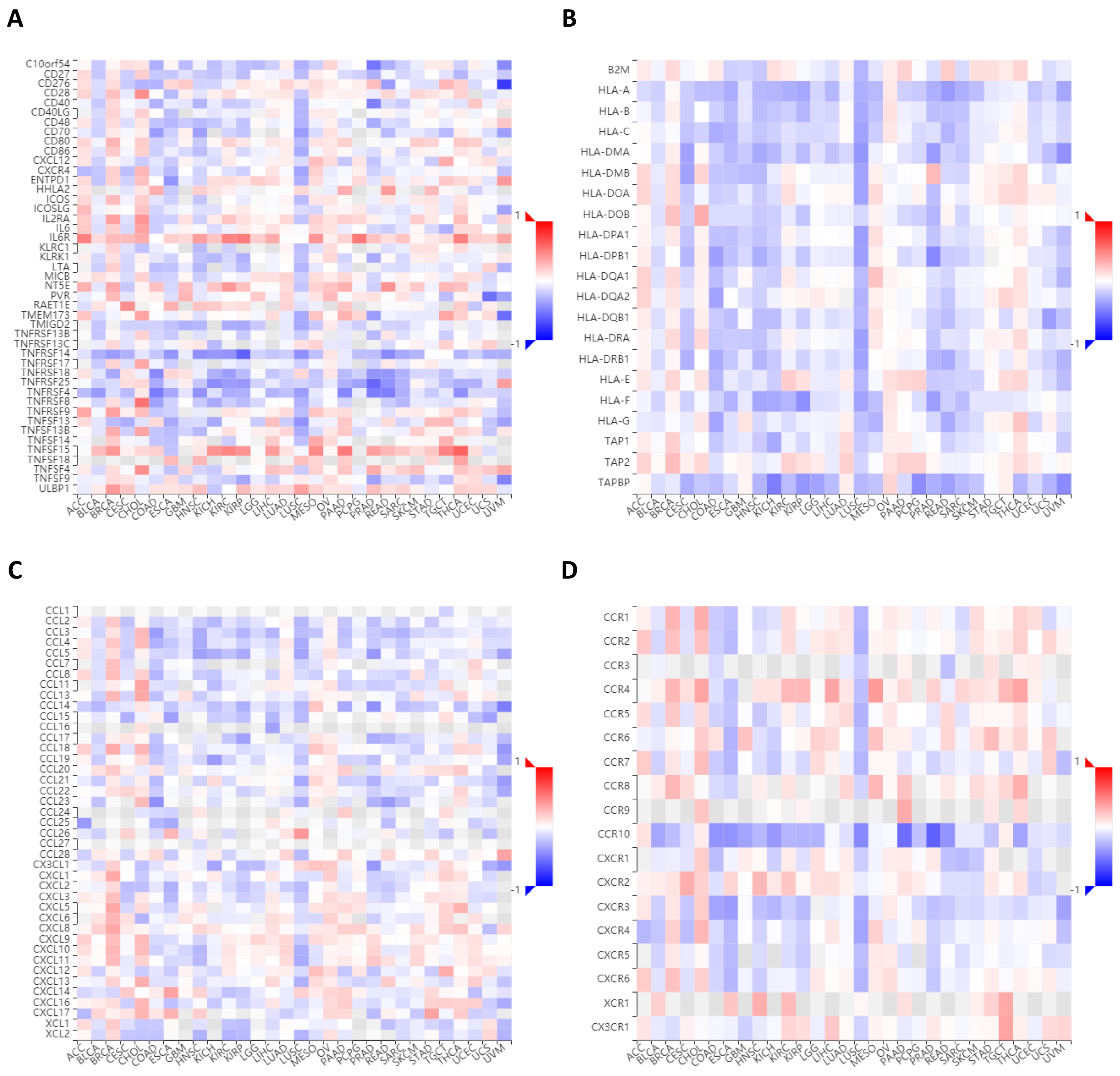
A
C10orf54
CD27
CD276
CD28
CD40
CD40LG
CD48
CD70
CD80
CD86
CXCL12
CXCR4
ENTPD1
HHLA2
ICOS
ICOSLG
IL2RA
IL6
IL6R
KLRC1
KLRK1
LTA
MICB
NT5E
PVR
RAET1E
TMEM173
TMIGD2
TNFRSF13B
TNFRSF13C
TNFRSF14
TNFRSF17
TNFRSF18
TNFRSF25
TNFRSF4
TNFRSF8
TNFRSF9
TNFSF13
TNFSF13B
TNFSF14
TNFSF15
TNFSF18
TNFSF4
TNFSF9
ULBP1
ACC BLCA BRCA CESC CHOL COAD ESCA GBM HNSC KICH KIRC KIRP LGG LIHC LUAD LUSC MESO OV PAAD PCPG PRAD READ SARC SKCM STAD TGCT THCA UCEC UCS UVM
1
-1
B
B2M
HLA-A
HLA-B
HLA-C
HLA-DMA
HLA-DMB
HLA-DOA
HLA-DOB
HLA-DPA1
HLA-DPB1
HLA-DQA1
HLA-DQA2
HLA-DQB1
HLA-DRA
HLA-DRB1
HLA-E
HLA-F
HLA-G
TAP1
TAP2
TAPBP
ACC BLCA BRCA CESC CHOL COAD ESCA GBM HNSC KICH KIRC KIRP LGG LIHC LUAD LUSC MESO OV PAAD PCPG PRAD READ SARC SKCM STAD TGCT THCA UCEC UCS UVM
1
-1
C
CCL1
CCL2
CCL3
CCL4
CCL5
CCL7
CCL8
CCL11
CCL13
CCL14
CCL15
CCL16
CCL17
CCL18
CCL19
CCL20
CCL21
CCL22
CCL23
CCL24
CCL25
CCL26
CCL27
CCL28
CX3CL1
CXCL1
CXCL2
CXCL3
CXCL5
CXCL6
CXCL8
CXCL9
CXCL10
CXCL11
CXCL12
CXCL13
CXCL14
CXCL16
CXCL17
XCL1
XCL2
ACC BLCA BRCA CESC CHOL COAD ESCA GBM HNSC KICH KIRC KIRP LGG LIHC LUAD LUSC MESO OV PAAD PCPG PRAD READ SARC SKCM STAD TGCT THCA UCEC UCS UVM
1
-1
D
CCR1
CCR2
CCR3
CCR4
CCR5
CCR6
CCR7
CCR8
CCR9
CCR10
CXCR1
CXCR2
CXCR3
CXCR4
CXCR5
CXCR6
XCR1
CX3CR1
ACC BLCA BRCA CESC CHOL COAD ESCA GBM HNSC KICH KIRC KIRP LGG LIHC LUAD LUSC MESO OV PAAD PCPG PRAD READ SARC SKCM STAD TGCT THCA UCEC UCS UVM
1
-1

Figure S8

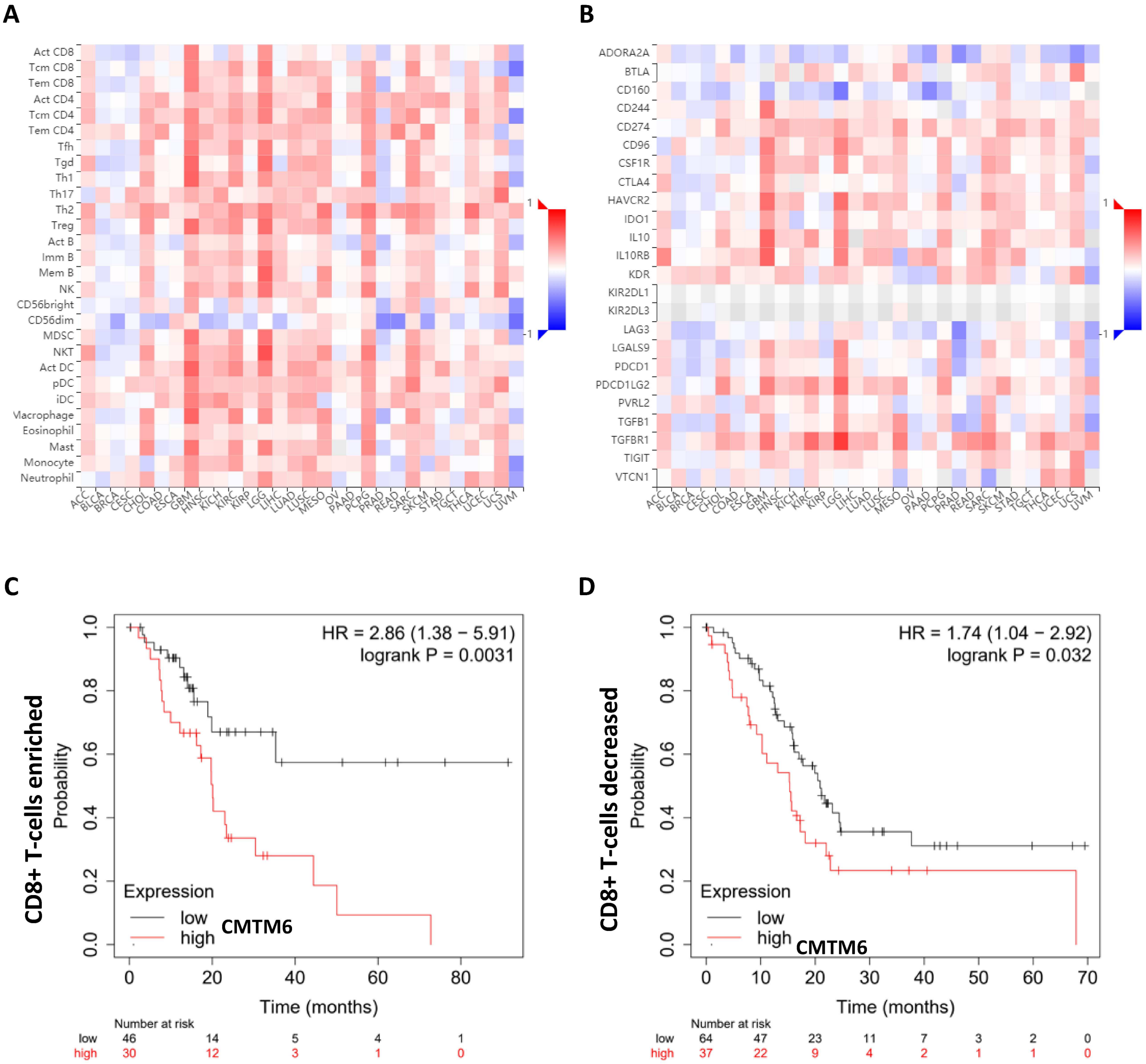
A
Act CD8
Tcm CD8
Tem CD8
Act CD4
Tcm CD4
Tem CD4
Tfh
Tgd
Th1
Th17
Th2
Treg
Act B
Imm B
Mem B
NK
CD56bright
CD56dim
MDSC
NKT
Act DC
pDC
iDC
Macrophage
Eosinophil
Mast
Monocyte
Neutrophil
ACC BLCA BRCA CESC CHOL COAD ESCA GBM HNSC KICH KIRC KIRP LGG LIHC LUAD LUSC MESO OV PAAD PCPG PRAD READ SARC SKCM STAD TGCT THCA UCEC UCS UVM
1
-1
B
ADORA2A
BTLA
CD160
CD244
CD274
CD96
CSF1R
CTLA4
HAVCR2
IDO1
IL10
IL10RB
KDR
KIR2DL1
KIR2DL3
LAG3
LGALS9
PDCD1
PDCD1LG2
PVRL2
TGFB1
TGFBR1
TIGIT
VTCN1
ACC BLCA BRCA CESC CHOL COAD ESCA GBM HNSC KICH KIRC KIRP LGG LIHC LUAD LUSC MESO OV PAAD PCPG PRAD READ SARC SKCM STAD TGCT THCA UCEC UCS UVM
1
-1
C
CD8+ T-cells enriched
Probability
HR = 2.86 (1.38 − 5.91)
logrank P = 0.0031
Expression
low
high
CMTM6
Time (months)
Number at risk
low 46 14 5 4 1
high 30 12 3 1 0
D
CD8+ T-cells decreased
Probability
HR = 1.74 (1.04 − 2.92)
logrank P = 0.032
Expression
low
high
CMTM6
Time (months)
Number at risk
low 64 47 23 11 7 3 2 0
high 37 22 9 4 2 1 1 0

Figure S9

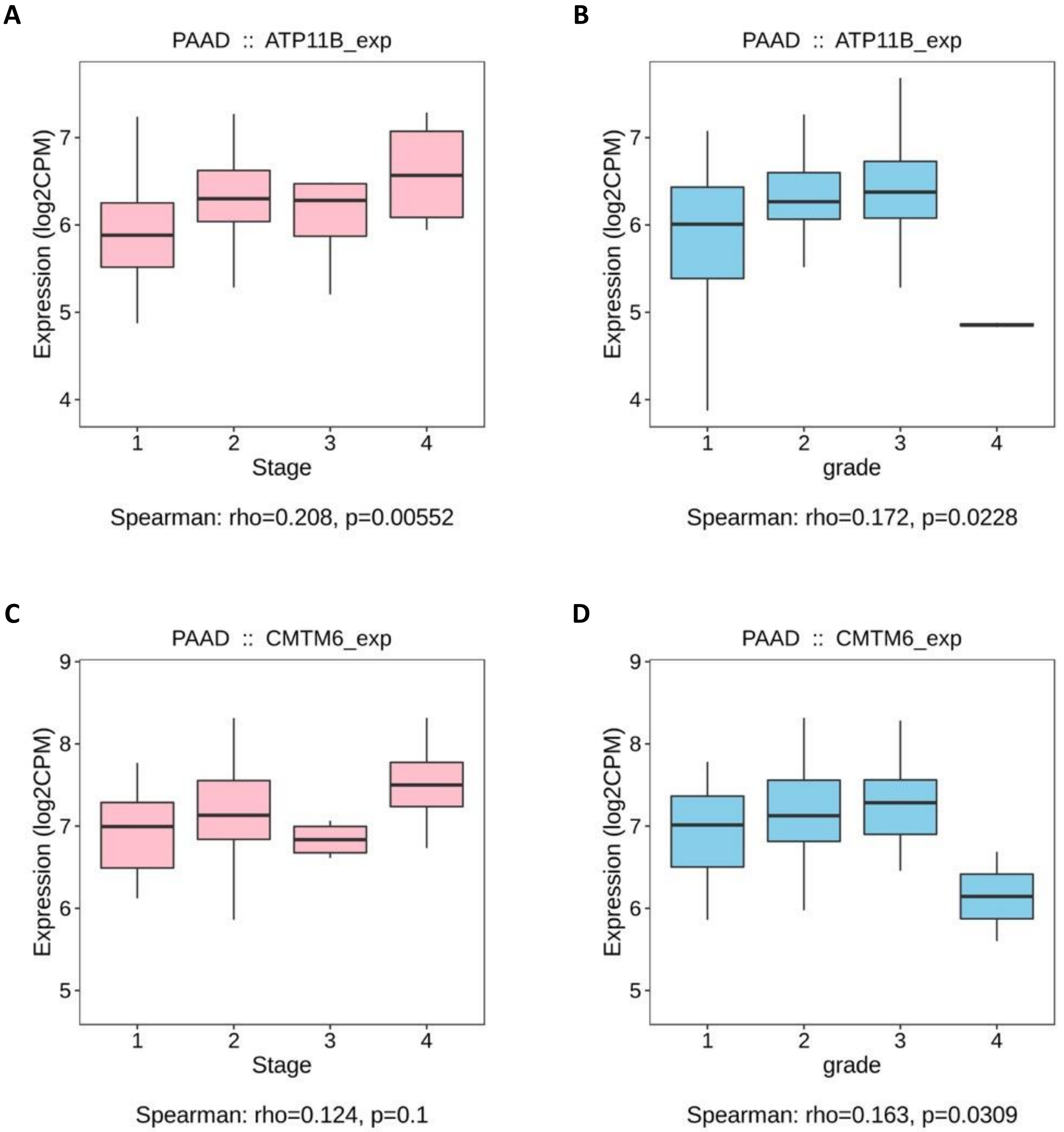
A
PAAD :: ATP11B_exp
Expression (log2CPM)
Stage
Spearman: rho=0.208, p=0.00552
B
PAAD :: ATP11B_exp
Expression (log2CPM)
grade
Spearman: rho=0.172, p=0.0228
C
PAAD :: CMTM6_exp
Expression (log2CPM)
Stage
Spearman: rho=0.124, p=0.1
D
PAAD :: CMTM6_exp
Expression (log2CPM)
grade
Spearman: rho=0.163, p=0.0309

Figure S10

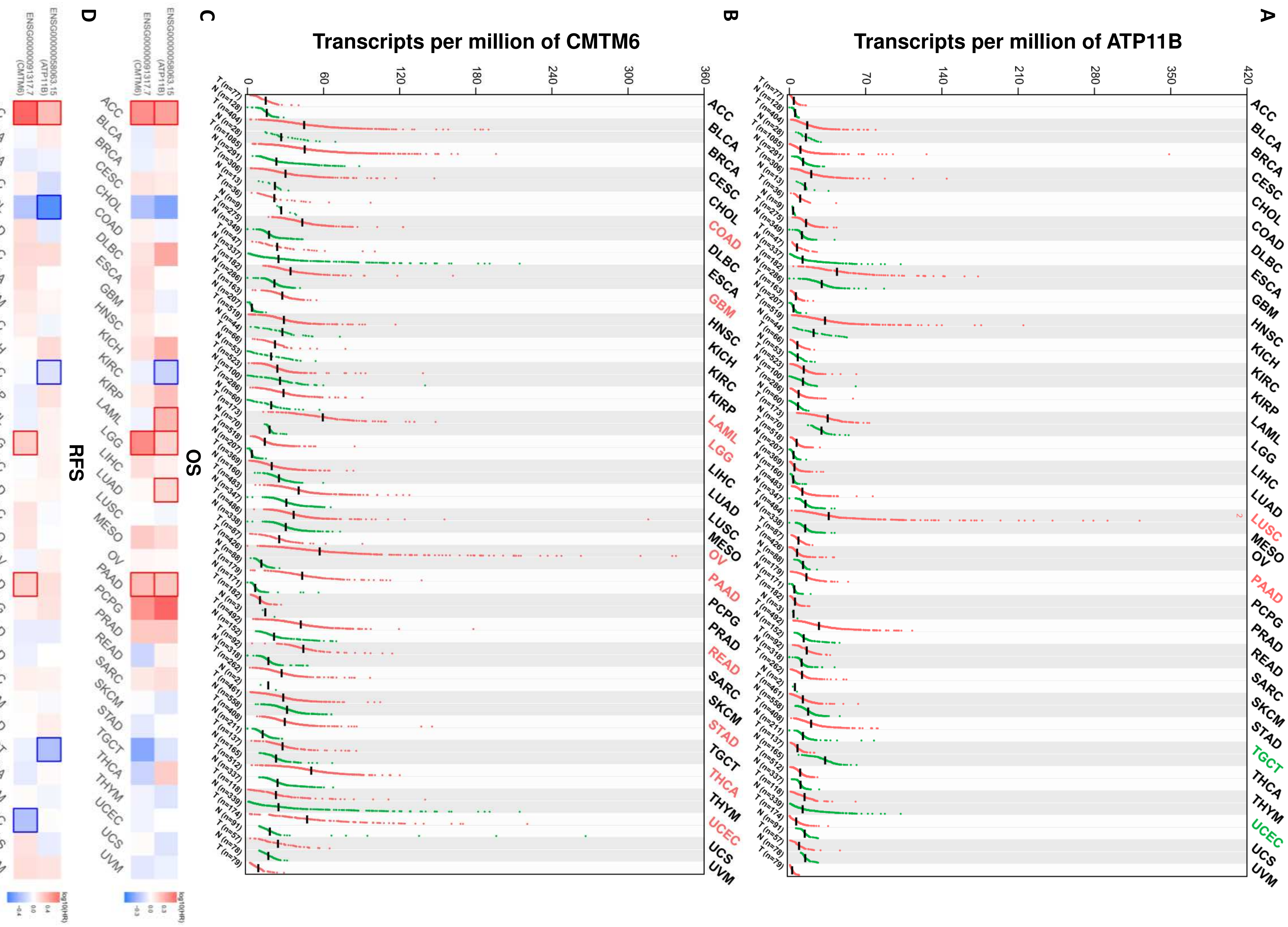
A
Transcripts per million of ATP11B
B
Transcripts per million of CMTM6
C
OS
D
RFS

Figure S11

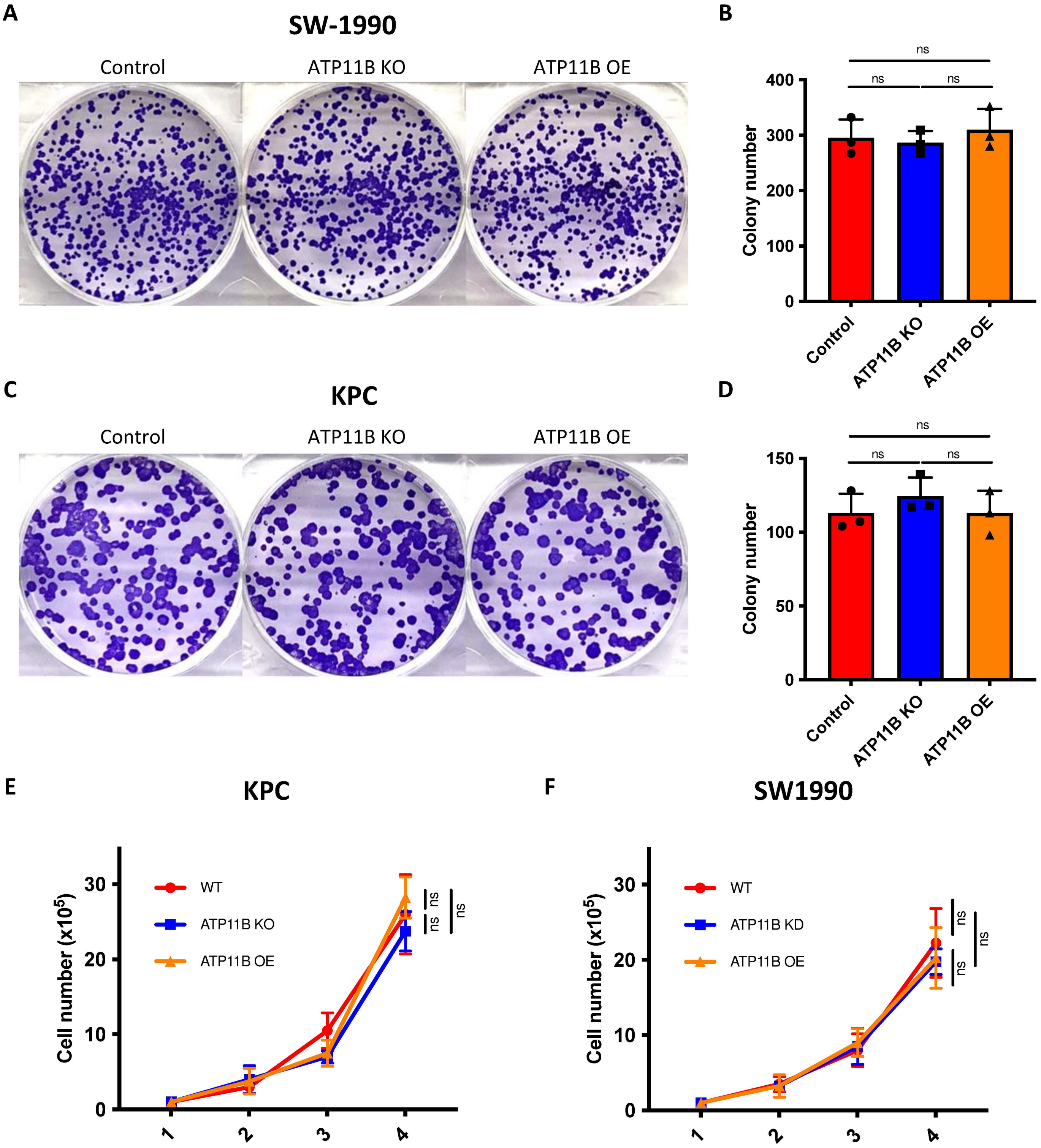
A
SW-1990
Control
ATP11B KO
ATP11B OE
B
ns
ns
ns
Colony number
0
100
200
300
400
Control
ATP11B KO
ATP11B OE
C
KPC
Control
ATP11B KO
ATP11B OE
D
ns
ns
ns
Colony number
0
50
100
150
Control
ATP11B KO
ATP11B OE
E
KPC
WT
ATP11B KO
ATP11B OE
Cell number (x10^5)
0
10
20
30
1
2
3
4
ns
ns
ns
F
SW1990
WT
ATP11B KD
ATP11B OE
Cell number (x10^5)
0
10
20
30
1
2
3
4
ns
ns
ns

Figure S12

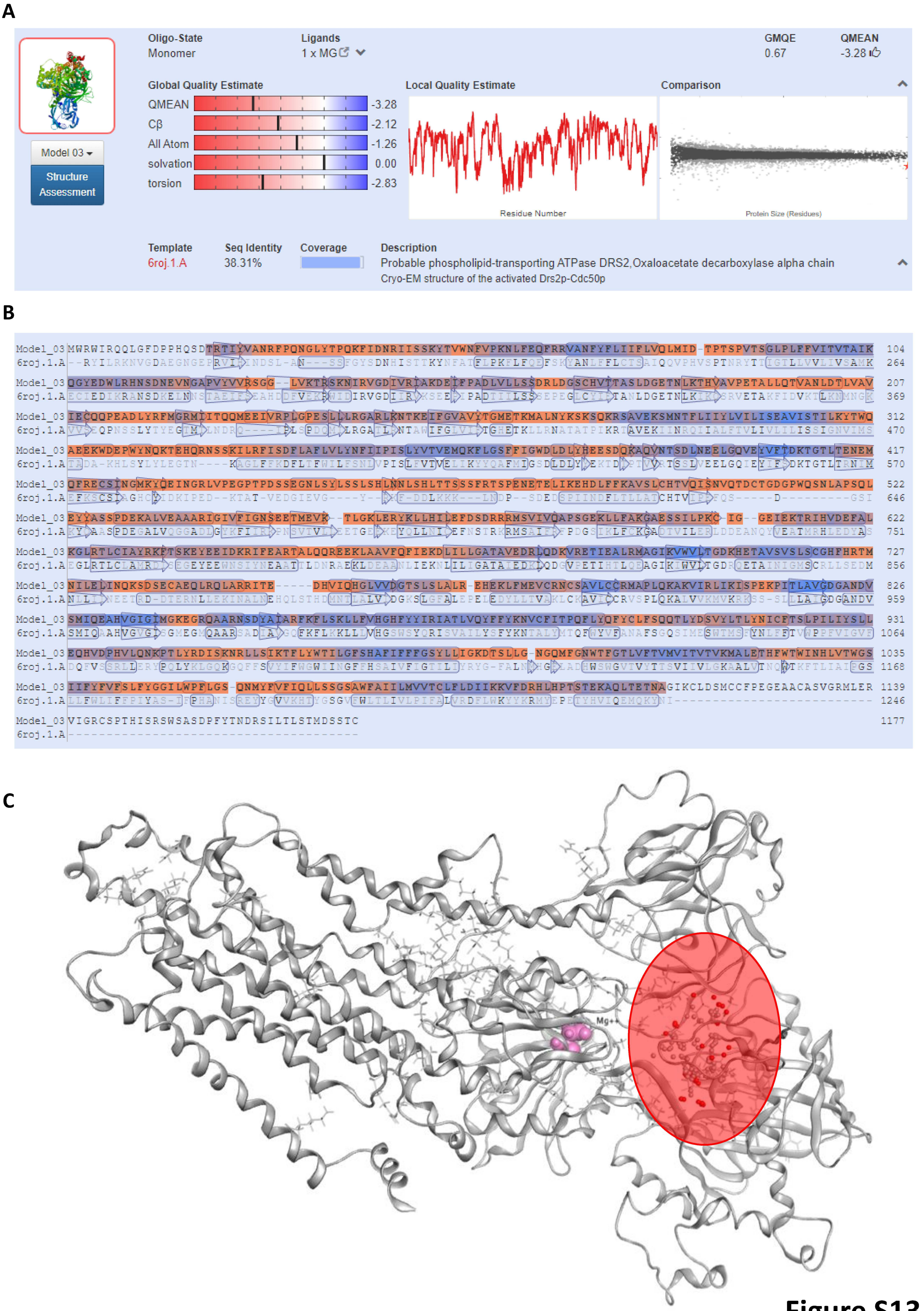
A
Oligo-State
Monomer
Ligands
1 x MG
GMQE
0.67
QMEAN
-3.28
Model 03
Structure Assessment
Global Quality Estimate
QMEAN -3.28
Cβ -2.12
All Atom -1.26
solvation 0.00
torsion -2.83
Local Quality Estimate
Residue Number
Comparison
Protein Size (Residues)
Template
6roj.1.A
Seq Identity
38.31%
Coverage
Description
Probable phospholipid-transporting ATPase DRS2,Oxaloacetate decarboxylase alpha chain
Cryo-EM structure of the activated Drs2p-Cdc50p
B
Model_03
6roj.1.A
C
Mg++

Figure S13

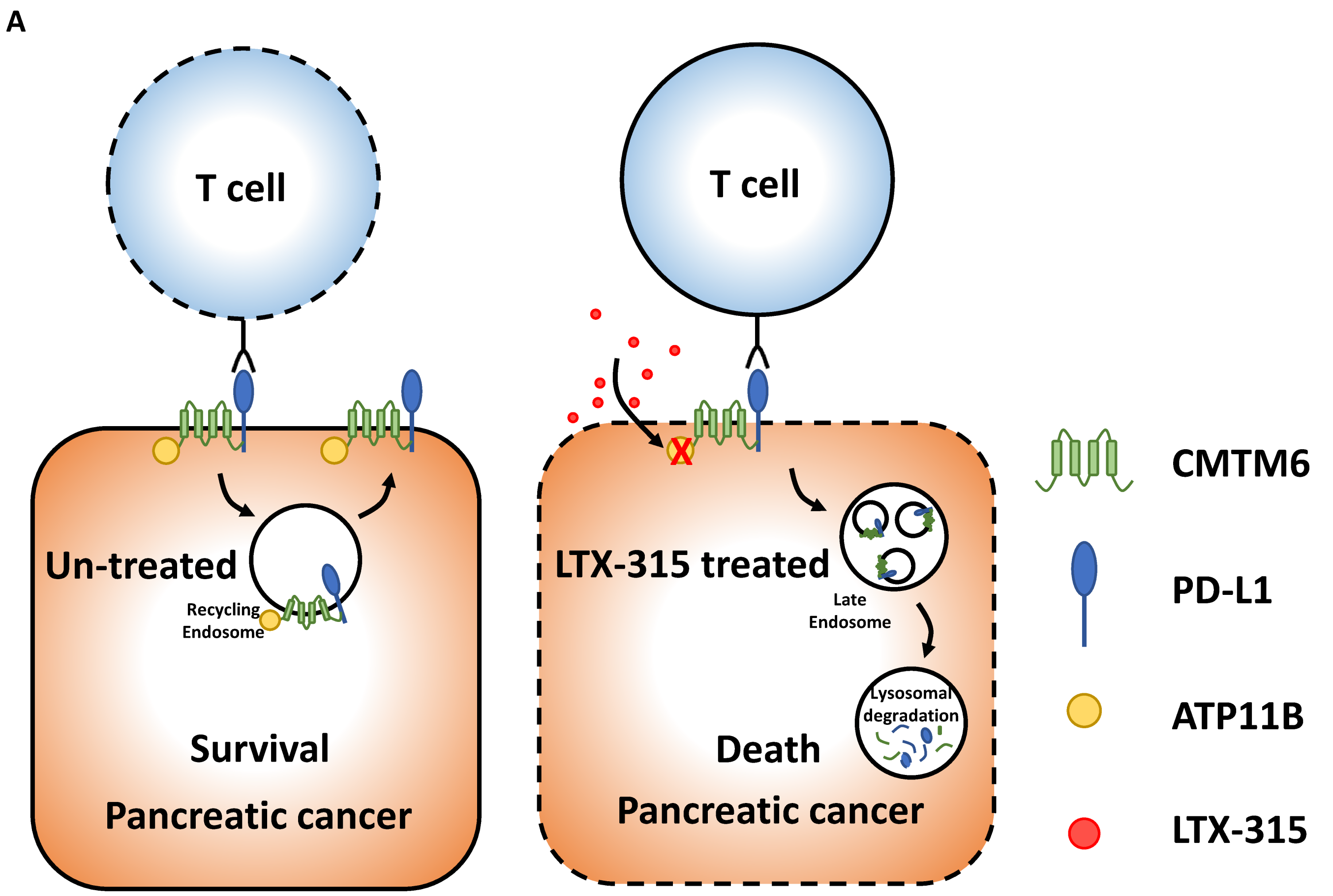
A
T cell
T cell
Un-treated
Recycling
Endosome
Survival
Pancreatic cancer
LTX-315 treated
Late
Endosome
Lysosomal
degradation
Death
Pancreatic cancer
CMTM6
PD-L1
ATP11B
LTX-315

Figure S14
