# Supplementary material for: Oncolytic peptide LTX-315 induces anti-pancreatic cancer immunity by targeting the ATP11B-PD-L1 axis
Source: J Immunother Cancer. 2022 Mar 14;10(3):e004129. doi: 10.1136/jitc-2021-004129 (PMC8921947; doi:10.1136/jitc-2021-004129)
Supplement: Supplementary data [file jitc-2021-004129supp002.pdf]

Supplementary figure legends

Figure S1. LTX-315 enhances the tumor infiltration of lymphocytes and prolongs mouse survival in combination with PD-L1-targeted therapy.

(A-B) Representative images of CD8 IHC staining (A) and further quantification (B). (C) Survival curves of mice treated with or without LTX-315 and PD-L1-targeted therapy. Time of death of mice treated with or without LTX-315 and PD-L1-targeted therapy individually recorded at the indicated times. Results are presented as mean ± SD of one representative experiment. **P* < 0.05, ***P* < 0.01, ****P* < 0.001 by a two-tailed *t*-test; ns: not significant.

Figure S2. LTX-315 synergizes with PD-L1-targeted therapy in hepatocellular carcinoma.

(A-E) Inhibition of hepatocellular carcinoma growth by the combination of LTX-315 and PD-L1-targeted therapy. Hepa1-6 cells s.c. inoculated into the immunocompetent mice (n=5) and growth curves of tumors recorded at the indicated time points (A). Representative images of tumor weight (B) and tumors (C) were individually recorded at the experimental endpoints. Representative images of TILs individually shown and further quantified (D-E).

Figure S3. Immune cell infiltration induced by LTX-315.

(A) KPC cells *s.c.* inoculated into the immunocompetent mice (n=6) treated with LTX-315. Tumors were collected at the indicated time and used for transcriptomics analysis. CIBERSORT was applied to quantify the infiltrated immune cells.

Tang T, *et al. J Immunother Cancer* 2022; 10:e004129. doi: 10.1136/jitc-2021-004129

Figure S4. Alteration in PD-L1 expression in the cell subsets of the tumor treated with LTX-315.

(A-B) Representative flow cytometry images of PD-L1 expression of tumor cells (A) and immune infiltrated cells (B) in tumors collected from immunocompetent mice treated with LTX-315.

Figure S5. Identification of LTX-315-regulated genes.

(A) Transcriptomics analysis reveals differentially expressed genes between KPC cells with or without LTX-315 treatment. Heat map of top-regulated genes by LTX-315.

Figure S6. LTX-315 alters IL-6 and IL-8 expression in pancreatic cancer cells.

(A-B) ELISA analysis of IL-6 and IL-8 in BXPC-3 (A) and SW1990 (B) cells treated with LTX-315.

Figure S7. LTX-315 downregulates ATP11B expression in pancreatic cancer.

(A-C) Inhibition of ATP11B expression by LTX-315 in a dose-dependent manner. ATP11B expression in BXPC-3 (A), SW1990 (B), and KPC (C) cells treated with LTX-315 at increasing concentrations, individually analyzed by western blotting. (D-F) Inhibition of ATP11B expression by LTX-315 in a time-dependent manner. ATP11B expression in BXPC-3 (D), SW1990 (E), and KPC (F) cell treated with LTX-315 at increasing time points, individually analyzed by western blotting. Results are presented as mean ± SD of one representative experiment. *$P < 0.05$, **$P < 0.01$, ***$P < 0.001$ by a two-tailed *t*-test; ns: not significant.

Figure S8. Immunological relevance of ATP11B in pancreatic cancer and other cancer types.

(A) Correlation between ATP11B and immunostimulators among multiple human cancers. (B) Correlation between ATP11B and MHCs among multiple human cancers. (C) Correlation between ATP11B and chemokines among multiple human cancers. (D) Correlation between ATP11B and chemokine receptors among multiple human cancers.

Figure S9. CMTM6 is associated with pancreatic cancer immunity.

(A) Correlation between CMTM6 expression and abundance of TILs among multiple human cancers. (B) Correlation between CMTM6 expression and immunoinhibitors among multiple human cancers. (C) OS analysis in pancreatic cancer patients with increased CD8+ T cells and low or high CMTM6 expression. (D) OS analysis in pancreatic cancer patients with decreased CD8+ T cells and low or high CMTM6 expression.

Figure S10. Association between ATP11B-CMTM6 axis and pancreatic cancer stage and grade.

(A) Association between ATP11B expression and stage in pancreatic cancer. (B) Association between ATP11B expression and grade in pancreatic cancer. (C) Association between CMTM6 expression and stage in pancreatic cancer. (D) Association between CMTM6 expression and grade in pancreatic cancer.

Tang T, *et al. J Immunother Cancer* 2022; 10:e004129. doi: 10.1136/jitc-2021-004129

Figure S11. The prognostic landscape of the ATP11B-CMTM6 axis in pancreatic cancer and other cancer types.

(A) Expression profile of ATP11B among multiple human cancers. (B) Expression profile of CMTM6 among multiple human cancers. (C) Contribution of ATP11B-CMTM6 axis to the OS among multiple human cancers. (D) Contribution of ATP11B-CMTM6 axis to disease-free survival (RFS) among multiple human cancers.

Figure S12. Effect of ATP11B on pancreatic cancer cell proliferation and colony formation.

(A-D) Representative images of colony formation of SW1990 (A) and KPC (C) cells with ATP11B depletion or overexpression, and further quantification (B, D). (E-F) The proliferation of KPC (E) and SW1990 (F) cells with ATP11B depletion or overexpression was individually recorded at the indicated time points.

Figure S13. Therapeutic potential of ATP11B as a target of small molecule inhibitors.

(A) Homology modeling of ATP11B by oxaloacetate decarboxylase alpha chain. (B) Model-template (6proj.1.A) alignment analysis. (C) 3D protein structure, active site, and small molecule binding pocket of ATP11B.

Figure S14. Hypothetical model representing the LTX-315 effect on the ATP11B-CMTM6-PD-L1 axis in pancreatic cancer immunity.

(A) A schematic model is proposed to illustrate how PD-L1 protein stability is regulated by ATP11B in pancreatic cancer. ATP11B interacts with PD-L1 in a

Tang T, *et al. J Immunother Cancer* 2022; 10:e004129. doi: 10.1136/jitc-2021-004129

CMTM6-dependent manner. Depletion of ATP11B results in the deregulation of CMTM6, leading to lysosome-mediated PD-L1 degradation.
